# Host-response trajectories in necrotizing fasciitis: host-pathogen interactions, immune evasion, and immunoparalysis

**DOI:** 10.3389/fcimb.2026.1874595

**Published:** 2026-07-01

**Authors:** Shuo Yang, Lin Jin, Lin Liu, Ling Wang, Zhiyong Hou

**Affiliations:** 1 Department of Orthopedic Surgery, The Third Hospital of Hebei Medical University, Shijiazhuang, Hebei, China; 2 Orthopaedic Research Institute of Hebei Province, Shijiazhuang, Hebei, China; 3 Department of Orthopedic Oncology, The Third Hospital of Hebei Medical University, Shijiazhuang, Hebei, China; 4 National Health Commission (NHC) Key Laboratory of Intelligent Orthopedic Equipment (The Third Hospital of Hebei Medical University), Shijiazhuang, Hebei, China; 5 Engineering Research Center of Orthopedic Minimally Invasive Intelligent Equipment, China Ministry of Education, Shijiazhuang, Hebei, China; 6 Key Laboratory of Biomechanics of Hebei Province, Shijiazhuang, Hebei, China; 7 Key Laboratory of Precise Assessment, Diagnosis, and Treatment of Soft Tissue Injury of Hebei Province, Shijiazhuang, Hebei, China

**Keywords:** group A streptococcus, host pathogen interaction, hyperinflammation, immune evasion, immunoparalysis, necrotizing fasciitis, necrotizing soft-tissue infection

## Abstract

Necrotizing fasciitis, a prototypical form of necrotizing soft-tissue infection, is a rapidly progressive and life-threatening soft-tissue infection characterized by extensive fascial necrosis, systemic toxicity, tissue hypoperfusion, septic shock, and multiorgan dysfunction. Although early recognition, urgent surgical source control, appropriate antimicrobial therapy, toxin suppression when indicated, and organ support remain the foundations of management, the dynamic host-response states that link microbial virulence, local tissue injury, impaired pathogen clearance, perfusion failure, and secondary immune dysfunction have not been fully integrated into a dedicated immunopathological framework. This Review conceptualizes necrotizing fasciitis as a pathogen-shaped, compartmentalized host-response trajectory in which toxin- and pattern-recognition-driven hyperinflammation, tissue hypoperfusion, impaired pathogen clearance, and counter-regulatory immune dysfunction may overlap across local and systemic compartments. Using group A Streptococcus as an illustrative pathogen, we summarize how superantigens, cytotoxins, immune-evasion programs, complement activation, neutrophil-driven injury, endothelial dysfunction, immunothrombosis, and hypoperfused tissue niches can amplify local destruction while limiting effective immune clearance. We further compare immunopathological differences between group A Streptococcus-predominant monomicrobial disease and polymicrobial infection, emphasizing how pathogen context, host susceptibility, tissue perfusion, and timing of source control may shape divergent clinical trajectories. Clinically relevant approaches for longitudinal host-response assessment are reviewed, including circulating biomarkers, monocytic HLA-DR expression, lymphocyte indices, functional immune profiling, and perfusion-oriented evaluation. Evidence derived from human necrotizing fasciitis or necrotizing soft-tissue infection cohorts is distinguished from mechanistic evidence based on invasive group A Streptococcus models and related sepsis literature. Host-directed immunomodulatory strategies remain largely investigational and should be interpreted through phenotype selection, timing, and evidence-boundary considerations. This host-response trajectory framework may help connect infection biology with immune monitoring, source-control reassessment, antimicrobial therapy, secondary infection surveillance, and phase-informed care. Prospective biomarker-stratified studies are needed to determine whether dynamic immune and perfusion phenotyping can improve survival, limb salvage, wound-bed readiness, reconstructive success, and long-term functional recovery.

## 1 Introduction

### 1.1 Disease overview: clinical severity and unmet burden

Necrotizing fasciitis (NF), a prototypical necrotizing soft-tissue infection, is a rapidly progressive, life-threatening infection marked by fascial necrosis, systemic toxicity, tissue hypoperfusion, and dysregulated inflammation (Hotchkiss et al., 2013; Stevens and Bryant, 2017; Brouwer et al., 2023). Despite resuscitation, antimicrobial therapy, urgent debridement, and intensive care, mortality commonly remains 20%-40% across cohorts and etiologies (Brouwer et al., 2023; Tedesco et al., 2025). Survivors often face limb loss, repeated debridement, complex wound management, reconstruction, prolonged intensive care, and persistent functional impairment. Geographic and seasonal variation in incidence, microbiology, and outcomes likely reflects pathogen ecology, host risk, environmental exposure, and healthcare access (Nelson et al., 2016; Nawijn et al., 2021). Clinically, severe pain out of proportion to skin findings, rapid edema or erythema, discoloration or crepitus, and early systemic deterioration reflect the convergence of microbial virulence, impaired perfusion, and maladaptive host immunity.

### 1.2 Etiologic heterogeneity and clinical context

NF is traditionally classified into four etiologic types: type I disease is polymicrobial, typically involving enteric Gram-negative bacilli and anaerobes; type II disease is usually monomicrobial and most often caused by Streptococcus pyogenes or other β-hemolytic streptococci; type III disease is associated with marine- or water-related pathogens such as Vibrio species; and type IV disease comprises fungal infections (Wong et al., 2003; Stevens and Bryant, 2017). These categories differ not only in microbiology but also in host predisposition, tissue environment, microbial virulence programs, and inflammatory context. Polymicrobial NF is often encountered in immunocompromised patients, perineal disease, postoperative wounds, chronic ulcers, or abdominal sources, whereas monomicrobial type II disease is more often associated with abrupt systemic toxicity, toxin-mediated amplification, and early shock physiology (Hakkarainen et al., 2014; Singh et al., 2016). This etiologic heterogeneity is clinically and immunologically important because host-response patterns, immune-monitoring signals, and the interpretation of potential adjunctive strategies cannot be separated from the underlying pathogen context.

### 1.3 Why an immune framework matters

As a leading cause of monomicrobial NF, group A Streptococcus (GAS) illustrates how microbial virulence interacts with host immunity to produce catastrophic tissue injury and systemic illness. GAS superantigens, cytotoxins, and immune-evasion programs can amplify cytokine release, endothelial injury, immunothrombosis, and hypoperfusion while impairing pathogen clearance (Hotchkiss et al., 2013; Stevens and Bryant, 2017; Brouwer et al., 2023). More broadly, NF is not only an infection requiring surgical source control but also a rapidly evolving host-response disorder within devitalized and hypoperfused tissue. Framing NF as a host-response disorder may help integrate operative findings, infection trajectory, tissue perfusion, and systemic immune signals during source-control reassessment, secondary-infection surveillance, organ-support decisions, and evidence-bounded evaluation of adjunctive strategies.

### 1.4 Host susceptibility and divergent trajectories

Host factors substantially influence susceptibility to NF and the tempo of disease progression. Major predisposing conditions include diabetes, peripheral vascular disease, immunosuppression, trauma, and recent surgery, although NF can also occur in previously healthy individuals (Hansen et al., 2017; Stevens and Bryant, 2017; Tedesco et al., 2025). Beyond these clinical risk factors, host genetic background, baseline immune tone, immunometabolic status, and local tissue perfusion may shape the magnitude, timing, and quality of the response to microbial toxins and inflammatory stimuli. These determinants likely contribute to marked interpatient heterogeneity, helping to explain why some patients develop fulminant toxin-amplified inflammation and early shock, whereas others show inadequate pathogen containment, earlier immune dysfunction, or impaired tissue repair.

### 1.5 Unmet need: tracking hyperinflammatory and immunosuppressive host-response states

A major unresolved problem in NF is how hyperinflammatory injury, impaired pathogen clearance, and secondary immune dysfunction are distributed across clinical time and anatomical compartments. Severe NF may contain concurrent host-response states: an intensely inflamed, pathogen-rich, and hypoperfused wound or fascial compartment can coexist with systemic lymphopenia, impaired antigen presentation, features of T-cell exhaustion, and increased vulnerability to secondary infection. We therefore use the “storm-to-silence” framework as a practical shorthand for a dynamic and compartmentalized host-response trajectory rather than as a fixed chronological sequence. In some patients, toxin- or pattern-recognition-amplified inflammation, characterized by fever, cytokine surge, neutrophilia, shock, endothelial injury, and microvascular failure, may overlap with or precede counter-regulatory immune dysfunction marked by lymphocyte apoptosis and myeloid deactivation, including depressed monocytic HLA-DR expression (Hotchkiss et al., 2013; Brouwer et al., 2023). These response states may differ between the wound bed, circulating immune cells, and organ compartments; nevertheless, this framework provides a practical lens for serial immune monitoring, source-control reassessment, secondary infection surveillance, and phase-informed critical care.

In this Review, we synthesize the major host-response mechanisms involved in NF, compare monomicrobial and polymicrobial immunopathology, summarize host determinants that shape disease trajectories, and discuss clinically relevant tools for dynamic immune and perfusion assessment. We further outline a phase-informed adjunctive framework that is intended to complement—rather than replace—the non-negotiable foundations of NF management: early surgical source control, appropriate antimicrobial therapy, and high-quality critical care support. Because direct NF-specific evidence remains limited for many immune-directed interventions, we distinguish evidence derived from human NF or NSTI cohorts from mechanistic and therapeutic concepts extrapolated from invasive GAS, sepsis immunology, and experimental host-response studies.

## 2 Immune phase transitions in necrotizing fasciitis

Immunologically, NF can be conceptualized as a dynamic host-response trajectory shaped by pathogen burden, tissue perfusion, source-control timing, and baseline host reserve. For conceptual clarity, we refer to two partially overlapping operational phenotypes. Phase I is dominated by pattern-recognition signaling and, in selected cases, superantigen-mediated T-cell activation, with complement activation, neutrophil/NET responses, cytokine release, endothelial injury, immunothrombosis, and microvascular dysfunction. Phase II involves lymphocyte apoptosis, low monocytic HLA-DR, myeloid deactivation, endotoxin tolerance-like features, and T-cell exhaustion, with risks of secondary infection, impaired healing, and viral reactivation.

**Figure 1:**
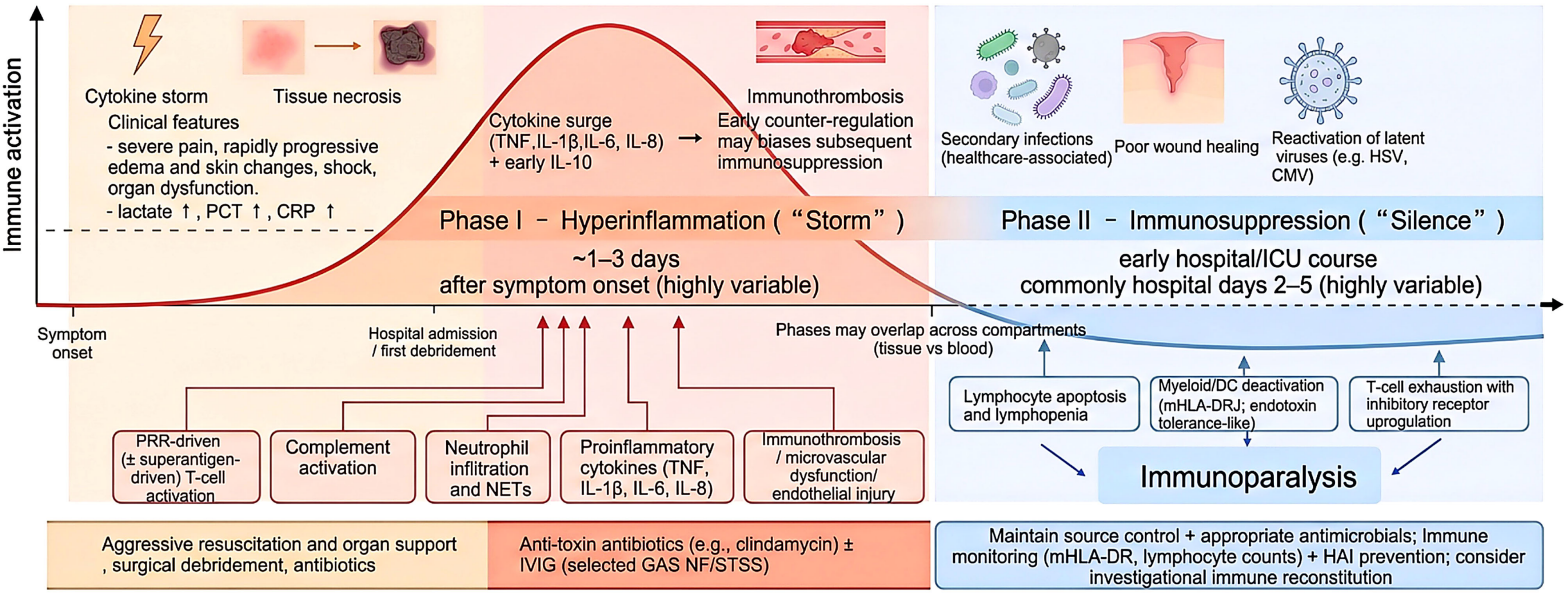
Biphasic temporal evolution of immune responses in necrotizing fasciitis: from hyperinflammatory “storm” to immunosuppressive “silence”. The schematic illustrates dominant but potentially overlapping immune-response patterns in necrotizing fasciitis (NF), including toxin- and pattern-recognition-amplified hyperinflammation and counter-regulatory immune dysfunction. Phase I is characterised by pathogen sensing, toxin-driven amplification, neutrophil recruitment/NET formation, complement activation, cytokine release, endothelial injury, and microvascular dysfunction, resulting clinically in rapidly progressive tissue necrosis, shock, and organ dysfunction. Phase II is characterised by lymphopenia, impaired antigen presentation, myeloid deactivation, and T-cell exhaustion, with increased vulnerability to secondary infection, delayed wound healing, and latent viral reactivation. These response patterns may overlap across tissue, blood, and organ compartments. The lower panel links this trajectory to monitoring and adjunctive-care priorities, while maintaining early source control, antimicrobial therapy, and organ support as the foundations of management.

**Figure 2:**
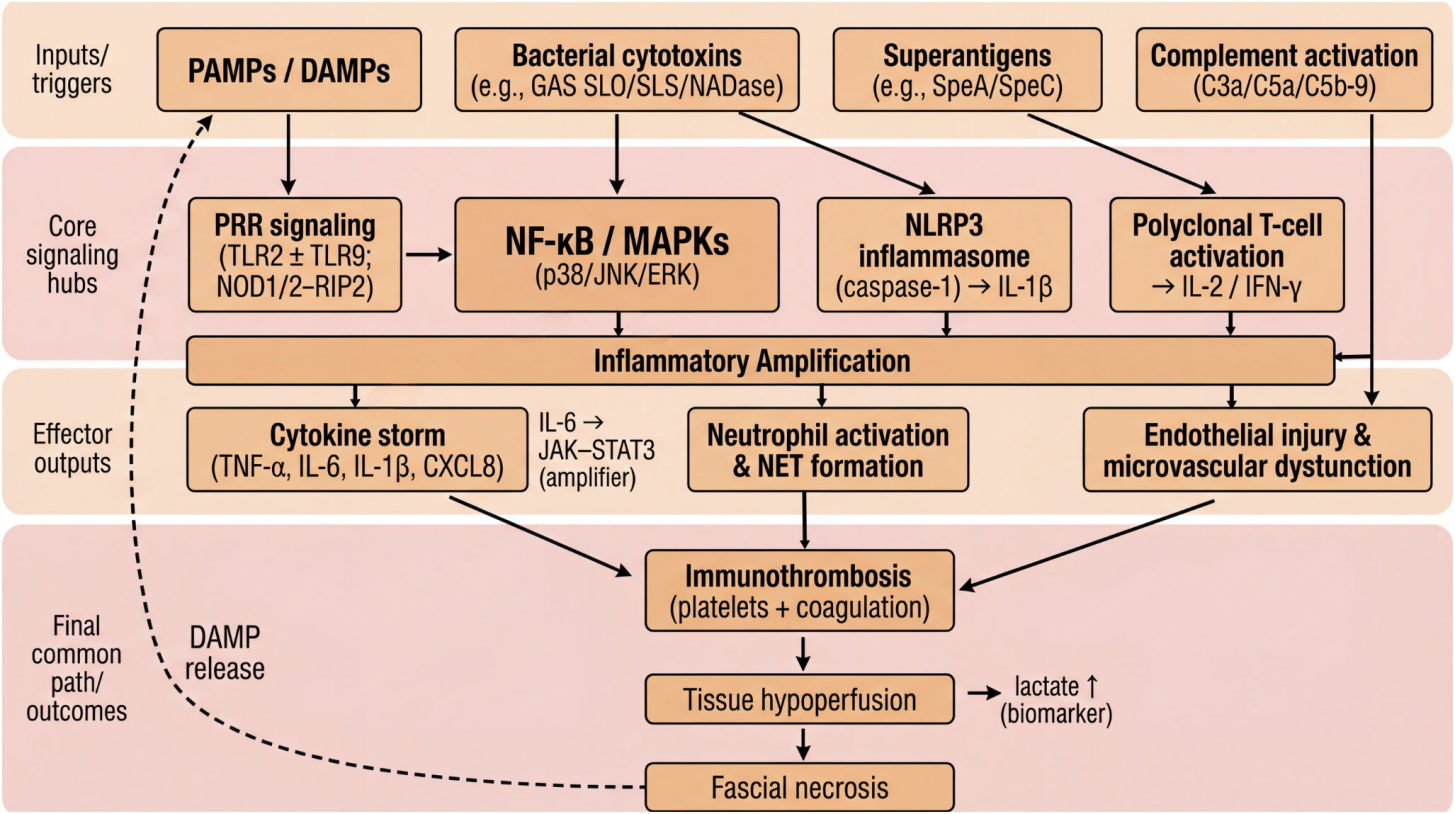
Phase I hyperinflammation (“storm”) in necrotizing fasciitis: from triggers to immunothrombosis and fascial necrosis. This image summarises the proposed immunopathogenic cascade during the early hyperinflammatory phase of NF. Microbial and host danger signals, bacterial cytotoxins, superantigens, and complement activation converge on pattern-recognition and inflammatory signalling pathways, including NF-κB/MAPK and inflammasome activation. These upstream events drive cytokine amplification, neutrophil activation/NET formation, endothelial injury, and immunothrombosis. The downstream consequences are tissue hypoperfusion, worsening fascial necrosis, and further danger-signal release, forming a feed-forward loop that sustains inflammatory injury.

**Figure 3:**
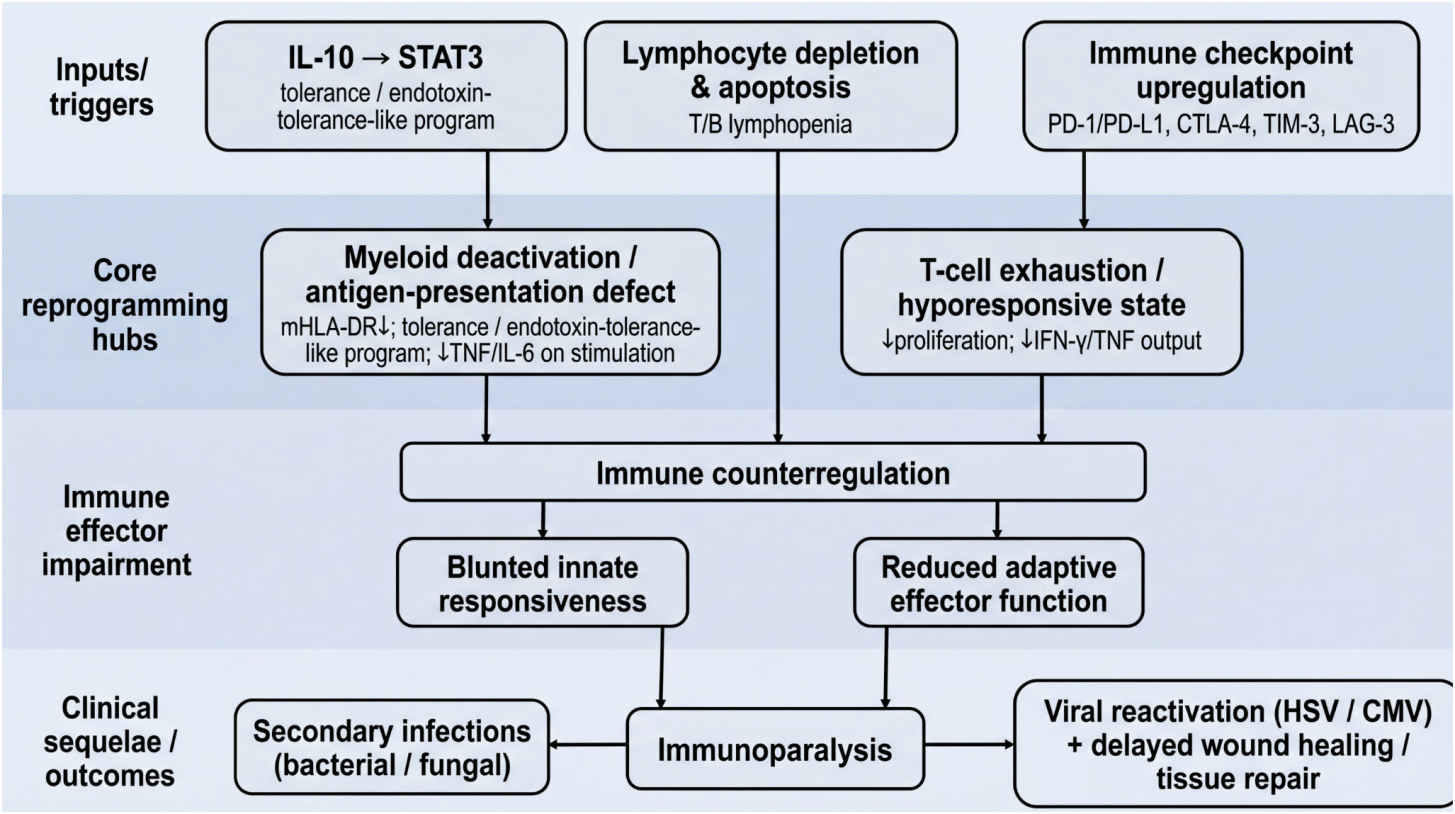
Phase II immunosuppression (“silence”) in necrotizing fasciitis: from immune counterregulation to immunoparalysis and secondary complications. The image outlines the major features of the later immunosuppressive phase of NF. Following early hyperinflammation, counter-regulatory pathways including IL-10–STAT3 signalling, lymphocyte depletion, and immune checkpoint upregulation contribute to myeloid deactivation and T-cell dysfunction. These changes are reflected by reduced antigen-presenting capacity, blunted innate responsiveness, and impaired adaptive effector function, culminating in immunoparalysis. Clinically, this state is associated with secondary bacterial or fungal infection, viral reactivation, delayed tissue repair, and poor wound healing.

These patterns should not be interpreted as fixed chronological stages or uniform whole-body states. In critically ill patients, immune responses may be spatially and temporally heterogeneous: the wound or fascial compartment may remain intensely inflamed, pathogen-rich, and hypoperfused, while circulating immune cells show lymphopenia, reduced mHLA-DR expression, impaired leukocyte responsiveness, or other features of immune deactivation. This local-systemic discordance is clinically important because peripheral biomarkers may not fully reflect the immune, microbial, or perfusion status of the wound bed. Accordingly, immune monitoring should be interpreted together with wound evolution, operative findings, microbiology, tissue perfusion, organ dysfunction, and source-control adequacy. **Figure 1** outlines this framework; **Figures 2** and **3** summarize Phase I and Phase II mechanisms.

Phase I generally refers to the early disease course of NF, often within approximately 1–3 days after symptom onset, although this timing is highly variable and influenced by pathogen burden, tissue perfusion, host susceptibility, and delay to source control. During this phase, resident and recruited innate immune cells mount a vigorous response to invasive pathogens within the fascia and subcutaneous tissues. This early response is essential for pathogen containment, but excessive or poorly compartmentalized activation can amplify cytokine release, endothelial injury, immunothrombosis, and tissue necrosis. This section delineates the early cascade that establishes the hyperinflammatory state.

### 2.1 Innate immune recognition

In NF, innate immunity shapes the early hyperinflammatory milieu. Bacterial PAMPs, including lipoteichoic acid, Gram-positive wall motifs, lipoproteins/lipopeptides, peptidoglycan fragments, and unmethylated CpG DNA, are sensed by PRRs on macrophages, dendritic cells, and neutrophils. TLR2 detects bacterial lipoproteins/lipopeptides and contributes to Gram-positive sensing; NOD1/2 recognize peptidoglycan fragments through RIP2; TLR9 detects CpG DNA; and murine TLR13 senses bacterial 23S rRNA (Schwandner et al., 1999; Li and Chen, 2012; Keestra-Gounder and Tsolis, 2017; Hafner et al., 2019).

The early immune response to GAS infection involves two coordinated processes. First, TLR–adaptor signaling, particularly through MyD88-dependent pathways, supports transcriptional priming of inflammatory mediators and inflammasome-related components, including TNF, IL-6, and pro-IL-1β. Second, toxin-associated inflammasome activation, exemplified by streptolysin O-mediated NLRP3 activation, promotes maturation and release of IL-1 family cytokines. Together, these processes amplify early cytokine responses, recruit and activate innate effector cells, and initiate the hyperinflammatory program characteristic of Phase I (Harder et al., 2009; Gratz et al., 2011; Castiglia et al., 2016; Hafner et al., 2019). The magnitude and persistence of this PRR-driven response depend on downstream signaling amplification, host counter-regulatory capacity, pathogen immune evasion, and the adequacy of early source control, as discussed in the following section.

### 2.2 Innate signaling cascades in early hyperinflammation

After PRR sensing, early inflammatory outputs are shaped by three interconnected signaling layers: transcriptional priming, inflammasome activation, and cytokine-receptor reinforcement. The canonical IKK–NF-κB axis provides the main transcriptional priming signal, inducing TNF, IL-6, pro-IL-1β, and inflammasome-related programs. In parallel, MyD88-dependent MAPK modules, including p38, JNK, and ERK, promote cytokine and chemokine production that supports leukocyte recruitment and endothelial activation (Pereira and Gazzinelli, 2023). In GAS infection, this priming layer can couple to streptolysin O-triggered NLRP3 inflammasome activation, thereby linking bacterial cytotoxicity to IL-1 family cytokine maturation (Harder et al., 2009). Human single-cell profiling of infected fascia has identified enrichment of NF-κB and PI3K–Akt pathways, supporting the activity of these networks in diseased tissue (Wang et al., 2025).

Cytokine-receptor signaling then reinforces this inflammatory network. IL-6 activates JAK–STAT3 and can cooperate with NF-κB-dependent programs, including the “IL-6 amplifier” in stromal and endothelial compartments (Hirano, 2021). PRR-triggered IRF programs induce type I interferons that modulate antimicrobial defense and leukocyte recruitment (Honda et al., 2006; Gratz et al., 2011). PI3K–Akt signaling may limit excessive cytokine production and promote compensatory IL-10 responses (Fukao et al., 2002; Polumuri et al., 2007), but sustained microbial stimulation, toxins, hypoperfusion, and delayed source control can overwhelm these counter-regulatory mechanisms. These observations support phenotype-selective immunomodulation as a research direction, but NF-specific evidence remains limited and anti-inflammatory strategies must be weighed against impaired pathogen clearance (Shakoory et al., 2016).

Functionally, these signaling networks induce key chemoattractants, including CXCL8/IL-8 and CXCL1, and endothelial adhesion molecules such as E-selectin, ICAM-1, and VCAM-1. The resulting chemokine-rich and endothelium-activated tissue environment facilitates the rapid neutrophil and monocyte influx described below.

### 2.3 Cellular recruitment and tissue infiltration

The chemokine-rich, endothelium-activated milieu recruits neutrophils and monocytes to fascia and subcutaneous tissue through CXCL8/IL-8, CXCL1/KC-like chemokines, and related mediators (Kolaczkowska and Kubes, 2013; Sawant et al., 2016; Cambier et al., 2023). Early NF lesions show polymorphonuclear infiltration in superficial fascia and deep subcutis, macrophage accumulation, and relative lymphocyte paucity, consistent with dominant innate immunity (Keller et al., 2018; Solomon et al., 2018). Experimental depletion studies support roles for neutrophils, macrophages, and dendritic cells in containment, although effects vary by pathogen, model, and depletion strategy (Loof et al., 2007; Hurst et al., 2022; Ozberk et al., 2023).

Neutrophils kill through phagocytosis, reactive oxygen species, degranulation, proteases, and NETs, but invasive GAS resists these defenses. The DNase Sda1 degrades NET DNA and facilitates escape from NET-mediated containment (Buchanan et al., 2006; Uchiyama et al., 2012). Resident innate cells also contribute; mouse studies suggest mast-cell cathelicidin-related peptides can limit GAS skin infection, although human NF relevance remains uncertain (Di Nardo et al., 2008). Thus, early innate recruitment both supports containment and, when excessive or counteracted by immune evasion, amplifies tissue injury.

### 2.4 Neutrophil-driven tissue injury and microvascular dysfunction

Although a vigorous inflammatory response can aid pathogen containment, excessive or poorly compartmentalized activation may inflict substantial collateral damage on host tissues. In early NF lesions, activated neutrophils release granule enzymes and proteases, including myeloperoxidase and neutrophil elastase, and generate reactive oxygen species, while activated macrophages contribute additional inflammatory mediators and matrix-remodeling programs. Together with high local concentrations of proinflammatory cytokines, these effector mechanisms promote tissue injury, vascular permeability, and endothelial dysfunction. During acute inflammation, neutrophil-derived proteases and oxidative bursts are key mediators of endothelial injury and tissue destruction (Korkmaz et al., 2010; Kolaczkowska and Kubes, 2013).

NF-related pathogens, particularly GAS, can further amplify this injury through toxin-associated inflammasome activation and IL-1 family signaling. For example, streptolysin O can trigger NLRP3 inflammasome activation, promoting caspase-1-mediated maturation of pro-IL-1β into biologically active IL-1β (Harder et al., 2009). IL-1β is a potent pyrogenic and proinflammatory cytokine that can intensify fever, leukocyte recruitment, and local inflammation. In concert with TNF, IL-1β enhances endothelial permeability and upregulates adhesion molecules, including E-selectin, ICAM-1, and VCAM-1, thereby facilitating leukocyte extravasation and reinforcing endothelial activation (Pober and Sessa, 2007).

Histologically, occlusive microvascular thrombi are frequent in NF lesions. NETs can provide prothrombotic scaffolds, linking neutrophil activation to immunothrombosis (Umbert et al., 1989; Fuchs et al., 2010). In GAS soft-tissue infection models, IL-1-driven neutrophil hyperactivation exacerbates muscle necrosis, hemorrhage, and microvascular dysfunction, creating a self-amplifying ischemia–inflammation cycle (Cullen et al., 2015). Microvascular thrombosis and endothelial dysfunction impair tissue perfusion, limit antimicrobial penetration and immune-effector access, and may contribute to progressive fascial necrosis despite systemic therapy. Sustained inflammatory and ischemic tissue injury may also coexist with, and potentially reinforce, systemic immune dysregulation, particularly when pathogen persistence, hypoperfusion, and delayed or incomplete source control maintain compartmentalized inflammatory stress.

### 2.5 Superantigen-driven T-cell activation and systemic inflammatory amplification

GAS can produce super antigenic exotoxins such as SpeA and SpeC, as well as SSA and SMEZ. These proteins bind MHC class II molecules and specific T-cell receptor Vβ regions, bypassing conventional antigen recognition and driving polyclonal T-cell activation (Spaulding et al., 2013). Rapid release of IFN-γ and IL-2 activates myeloid TNF, IL-1, IL-6, and other mediators. In severe GAS disease, this circuit contributes to capillary leak, shock, and multiorgan dysfunction typical of STSS (Lappin and Ferguson, 2009). Because prior antigen-specific sensitization is unnecessary, previously healthy individuals can deteriorate abruptly (Lappin and Ferguson, 2009; Spaulding et al., 2013).

Host immunogenetic background may further tune the intensity of this superantigen axis. HLA class II variation has been associated with differences in susceptibility to severe invasive GAS disease and with allele-dependent variability in superantigen binding, presentation, and downstream T-cell cytokine release (Llewelyn et al., 2004; Llewelyn, 2005; Parks et al., 2020). Thus, under similar pathogen exposures, differences in host antigen-presentation context may contribute to divergent degrees of superantigen-driven inflammatory amplification and clinical severity. This host-genetic layer is considered further in Section 4.1 within the broader discussion of baseline immune reserve.

In summary, the superantigen–T-cell axis can act as a potent amplifier that rapidly escalates systemic inflammatory stress, endothelial dysfunction, and microcirculatory failure on top of upstream PRR-driven innate activation. Superantigen-elicited cytokine bursts can reinforce intracellular signaling networks and engage downstream humoral and cellular amplification loops, accelerating mediator release and tissue injury. This framework sets the stage for discussing how pre-existing antitoxin immunity and other humoral factors may modulate the intensity of superantigen-driven disease.

### 2.6 Humoral modulation of the superantigen axis

Pre-existing toxin- and superantigen-neutralizing antibodies may modulate GAS-driven inflammation by reducing extracellular virulence-factor activity and downstream T-cell activation. Many adults have anti-streptolysin O or anti-DNase B antibodies, and antibodies to surface antigens such as M protein can support opsonophagocytic clearance (Delice et al., 2015; Tsoi et al., 2015; Brimo et al., 2021). Prior exposure, however, does not ensure neutralization of disease-relevant toxins. Neutralizing anti-SLO antibodies reduce cytolysis *in vitro* (Sierig et al., 2003; Bi et al., 2022), and absent neutralizing activity against SpeA has been linked to severe invasive GAS phenotypes (Darenberg et al., 2003; Linner et al., 2014). B-cell-intrinsic roles beyond antibody production in acute NF remain poorly defined (Rastogi et al., 2022). IVIG implications are discussed later in the treatment section.

Beyond antibody-mediated neutralization, extracellular humoral cascades, most notably complement, can function as rapid amplifiers of inflammation and microvascular injury during Phase I. This complement-mediated amplification is discussed below.

### 2.7 Complement activation and inflammatory mediator amplification

Complement can intensify Phase I hyperinflammation and microvascular injury. Microbial surfaces, immune complexes, and DAMPs activate classical, lectin, and alternative pathways, generating C3b, C3a, C5a, and C5b-9. These effectors support opsonophagocytic clearance and leukocyte recruitment, but excessive activation may be harmful. In NSTI cohorts, admission complement activation markers, including C4c/C4 and C3bc/C3 ratios, have predicted 90-day mortality, while higher C4d and soluble C5b-9 have been associated with greater severity and 30-day mortality (Kristensen et al., 2020; Hedetoft et al., 2022). Broader sepsis data suggest that sustained C5a signaling may promote neutrophil dysfunction, endothelial permeability, and vascular dysregulation, whereas C5b-9 can induce proinflammatory and procoagulant endothelial phenotypes (Yan and Gao, 2012; Fishelson and Kirschfink, 2019). These findings support complement activation as a severity-linked pathway, but not as a validated therapeutic target in NF.

Phase I is also marked by elevated circulating mediators, especially in GAS-associated disease. In 159 NSTI patients, septic shock was associated with higher IL-1β, IL-6, IL-10, and TNF-α; IL-6 correlated with organ failure, whereas IL-1β and IL-10 predicted 30-day mortality (Hansen et al., 2017). Larger studies identify IL-6 as a recurring shock marker and report differences in IL-10, IL-11, and MMP-9 between monomicrobial and polymicrobial disease (Palma et al., 2021). GAS-associated NF shows higher CXCL8/IL-8, IL-1β, and TNF-α than non-necrotizing infection (Rath et al., 2023). TNF and IL-1β promote vasodilation, endothelial junction disruption, and capillary leak, whereas early IL-10 may shape later immune trajectories (Hotchkiss et al., 2013; Hansen et al., 2017; Jarczak et al., 2021).

In summary, Phase I in NF reflects a dysregulated convergence of intracellular inflammatory signaling with extracellular humoral and soluble-mediator amplification circuits. Dominant proinflammatory programs can accelerate endothelial injury, immunothrombosis, tissue hypoperfusion, and microcirculatory collapse, whereas counter-regulatory mediators may emerge in parallel and contribute to immune heterogeneity across tissue and systemic compartments (Hotchkiss et al., 2013; Hansen et al., 2017). Thus, hyperinflammatory injury and immune-suppressive features should be viewed as potentially overlapping response patterns whose relative dominance depends on pathogen burden, tissue perfusion, source-control timing, and baseline host reserve.

### 2.8 Phase II: immune counter regulation and secondary suppression

During severe NF, immune counter-regulation and secondary immune dysfunction may become clinically evident during the early ICU course, particularly after the initial inflammatory insult, resuscitation, operative debridement, and antimicrobial treatment have begun. This state should not be equated with disappearance of local inflammation or complete control of infection. Peripheral immune dysfunction may develop in parallel with a persistently inflamed, pathogen-rich, or hypoperfused wound bed, especially when tissue necrosis, microbial burden, or incomplete source control continue to drive compartmentalized inflammatory stress. Its timing and intensity depend on symptom duration before presentation, pathogen burden, adequacy of debridement, antimicrobial efficacy, tissue perfusion, baseline host immunity, and ICU exposures.

Operationally, Phase II refers to a host-response pattern characterized by lymphocyte depletion, reduced monocytic HLA-DR expression with impaired antigen-presenting capacity, myeloid deactivation, endotoxin tolerance-like features, and T-cell functional exhaustion. These features may coexist with ongoing wound inflammation, organ dysfunction, or residual infection rather than replacing them. Recognition of this pattern therefore requires integrated interpretation of peripheral immune markers, clinical trajectory, wound evolution, microbiology, tissue perfusion, and source-control adequacy.

The emergence of immune suppression requires continued reassessment of source control and antimicrobial adequacy, close surveillance for secondary infections, and careful avoidance of unnecessary iatrogenic immunosuppression. Where immune monitoring is available, serial markers such as persistent lymphopenia, delayed recovery of mHLA-DR expression, or impaired ex vivo leukocyte responsiveness may help enrich future studies of immune-restorative approaches, including GM-CSF-, IFN-γ-, IL-7-, or checkpoint-oriented strategies discussed in Section 6.2.2. The following sections discuss the major components of this state, including lymphocyte loss, impaired antigen presentation, myeloid deactivation, and T-cell exhaustion.

#### 2.8.1 Lymphocyte depletion and apoptosis

Declining circulating lymphocytes, including CD4+ and CD8+ T cells, are measurable features of secondary immune dysfunction in severe infection and may characterize Phase II NF. Lymphopenia reflects apoptosis, altered trafficking, impaired proliferative recovery, host factors, critical illness, and treatments. B-cell lymphopenia may parallel T-cell loss, although B-cell-specific functions in acute NF remain unclear (Boomer et al., 2011; van der Poll et al., 2017; Leijte et al., 2020). Persistent lymphopenia is associated with secondary/opportunistic infections and impaired repair in severe infection (Boomer et al., 2011; Hotchkiss et al., 2013; van der Poll et al., 2017; Leijte et al., 2020).

#### 2.8.2 IL-10–STAT3 signaling, endotoxin tolerance-like reprogramming, and mHLA-DR suppression

A key immunological alteration during secondary immune dysfunction is reduced monocytic HLA-DR (mHLA-DR) expression in peripheral blood, reflecting impaired antigen-presenting capacity. Upon ex vivo stimulation, for example with lipopolysaccharide, monocytes from critically ill patients often show a reduced ability to produce proinflammatory cytokines such as TNF-α and IL-6, resembling an immune-reprogramming phenotype analogous to endotoxin tolerance described after severe sepsis (Boomer et al., 2011; Hotchkiss et al., 2013; van der Poll et al., 2017; Leijte et al., 2020). In NF, persistent reduction of mHLA-DR, particularly when accompanied by lymphopenia or impaired leukocyte responsiveness, may indicate an immunoparalysis-like state. This functional inhibition is shaped by multiple counter-regulatory pathways, among which IL-10–STAT3 signaling represents an important axis.

IL-10 deactivates monocytes/macrophages largely through JAK1-TYK2-STAT3. STAT3 induces SOCS proteins, SHIP1, DUSP1, and miR-146b, dampening TLR- and cytokine receptor-proximal signaling (Curtale et al., 2013). IL-10-STAT3 also limits NF-kappaB/MAPK activation, suppresses MHC class II expression through MARCH1-associated turnover, and reduces co-stimulation (Kontoyiannis et al., 2001; Mittal et al., 2015). These mechanisms support mHLA-DR-low monocytes and blunted cytokine output, although inflammation, metabolism, stress hormones, and epigenetic reprogramming also contribute.

Clinical studies in sepsis and related critical illness have shown that persistently low mHLA-DR expression during the first week of hospitalization or ICU care is associated with increased risk of secondary infections and mortality, with delayed recovery more often observed in non-survivors (Monneret et al., 2006; Biswas and López-Collazo, 2009; Guignant et al., 2011; Leijte et al., 2020; Xia et al., 2023). Accordingly, in centers where immune monitoring is available, serial mHLA-DR measurements, often interpreted alongside lymphocyte counts, cytokine patterns, and functional stimulation assays, may help identify patients with immunoparalysis-like features. In NF, such patients may represent a rational population for future biomarker-stratified studies of immune-restorative interventions, provided that adequate source control and antimicrobial therapy have been secured.

#### 2.8.3 Immune checkpoint-associated T-cell dysfunction

Phase II may include exhaustion-like T-cell dysfunction. In severe infection, surviving T cells can upregulate PD-1, CTLA-4, TIM-3, and LAG-3, with reduced proliferation and IFN-γ/TNF-α production (Boomer et al., 2011; Guignant et al., 2011; van der Poll et al., 2017). PD-1-PD-L1 engagement dampens TCR/CD28 signaling through phosphatases such as SHP2. By analogy with sepsis, severe NF may develop adaptive hyporesponsiveness when lymphopenia, antigenic stimulation, inflammation, and counter-regulation coexist, potentially impairing secondary-infection control.

#### 2.8.4 Myeloid deactivation and dendritic-cell dysfunction

Phase II is also marked by functional reprogramming of monocytes, macrophages, and dendritic cells toward a deactivated or tolerance-like state. IL-10–STAT3 signaling contributes importantly to this process by promoting anti-inflammatory and regulatory programs, including increased IL-10 and TGF-β production, reduced TNF-α and IL-12 responses, and attenuation of antigen-presenting and co-stimulatory functions (Xia et al., 2023). However, myeloid deactivation is not attributable to a single pathway; sustained inflammatory exposure, metabolic stress, neuroendocrine signals, epigenetic remodeling, and persistent microbial or tissue-derived stimuli may also shape this phenotype.

Functionally, myeloid deactivation includes reduced mHLA-DR, blunted stimulated cytokine production, impaired chemotaxis or phagocytosis, and inhibitory programs in an anti-inflammatory milieu (Boomer et al., 2011; Guignant et al., 2011; van der Poll et al., 2017; Leijte et al., 2020). In sepsis, persistent low mHLA-DR and poor leukocyte responsiveness after stabilization associate with secondary infection and adverse outcomes (Monneret et al., 2006; Biswas and López-Collazo, 2009; Guignant et al., 2011; Venet and Monneret, 2018; Xia et al., 2023). In NF, analogous dysfunction after source control may justify intensified infection surveillance and future immune-restorative trials.

#### 2.8.5 Clinical sequelae of immunoparalysis: secondary infection and impaired repair

Secondary immune dysfunction creates vulnerability to healthcare-associated infection, delayed repair, and, in selected critically ill patients, viral reactivation or fungal superinfection. Ventilator-associated pneumonia and catheter-related bloodstream infection may occur in the setting of immunoparalysis and ICU exposure. Wound repair may also lag because impaired immune coordination, residual microbial burden, hypoperfusion, and microvascular dysfunction hinder clearance of debris and necrotic material. HSV and CMV reactivation are reported in critical illness, although NF-specific evidence is limited (Limaye et al., 2008; Osawa and Singh, 2009; Walton et al., 2014; van der Poll et al., 2017).

Recognition of an immunoparalysis-like trajectory is therefore important for adapting monitoring and supportive care without compromising infection control. Source-control adequacy and antimicrobial therapy must continue to be reassessed, while the risk–benefit profile of any host-directed adjunct should be interpreted in light of diminished immune responsiveness. Clinical management should prioritize intensified surveillance for secondary infections, strict adherence to infection-prevention practices, minimization of unnecessary iatrogenic immunosuppression, and immune-monitoring-guided identification of patients who may be suitable for future biomarker-stratified studies of immune-restorative strategies (Guignant et al., 2011; van der Poll et al., 2017; Venet and Monneret, 2018).

#### 2.8.6 Immune recovery and rationale for biomarker-guided immune restoration

Once adequate source control has been achieved and ongoing invasive infection is no longer the dominant clinical driver, recovery from an immunoparalysis-like state may be reflected by rising lymphocyte counts, restoration of mHLA-DR expression, and gradual improvement in leukocyte responsiveness. Conversely, persistent lymphopenia, sustained mHLA-DR depression, impaired ex vivo leukocyte responses, or recurrent secondary infection may indicate ongoing immune dysfunction after the initial hyperinflammatory insult.

This concept provides the biological rationale for biomarker-guided immune-restorative research, but it does not establish immune stimulation as standard care in NF. Candidate approaches, including GM-CSF, IFN-γ, IL-7, checkpoint-based modulation, and selected strategies targeting suppressive pathways, have been explored mainly in sepsis-associated immunoparalysis, preclinical models, or early-phase translational settings rather than in NF-specific trials (Docke et al., 1997; Meisel et al., 2009; Bah et al., 2018; Francois et al., 2018; Hotchkiss et al., 2019). Their potential application in NF therefore requires objective immune-dysfunction enrichment, adequate source control, predefined safety criteria, and prospective validation, as discussed further in Section 6.2.2.

In survivors, immune recovery should also be interpreted in the broader context of wound healing, metabolic and nutritional status, rehabilitation, and prevention of recurrent soft-tissue infection. No licensed Strep A vaccine is currently available, and leading candidates, including multivalent M protein-based formulations and J8-based peptide vaccines, remain in clinical development (Pastural et al., 2020; Meier-Stephenson et al., 2024). Routine long-term antibiotic prophylaxis after recovery from NF or invasive GAS disease is not standard practice and, when considered, generally falls within public health guidance for selected high-risk contacts or specific risk contexts (Durand et al., 2013).

**Figures 2** and **3** summarize the Phase I and Phase II mechanisms that shape the “storm-to-silence” trajectory. Phase II immunosuppression represents counter-regulation rather than resolution of inflammation. Because pathogen persistence in hypoperfused niches may prolong injury and dysfunction, the next section examines GAS immune evasion across this trajectory.

### 2.9 Pathogen immune evasion mechanisms: sculpting host responses and promoting persistence

**Figure 4:**
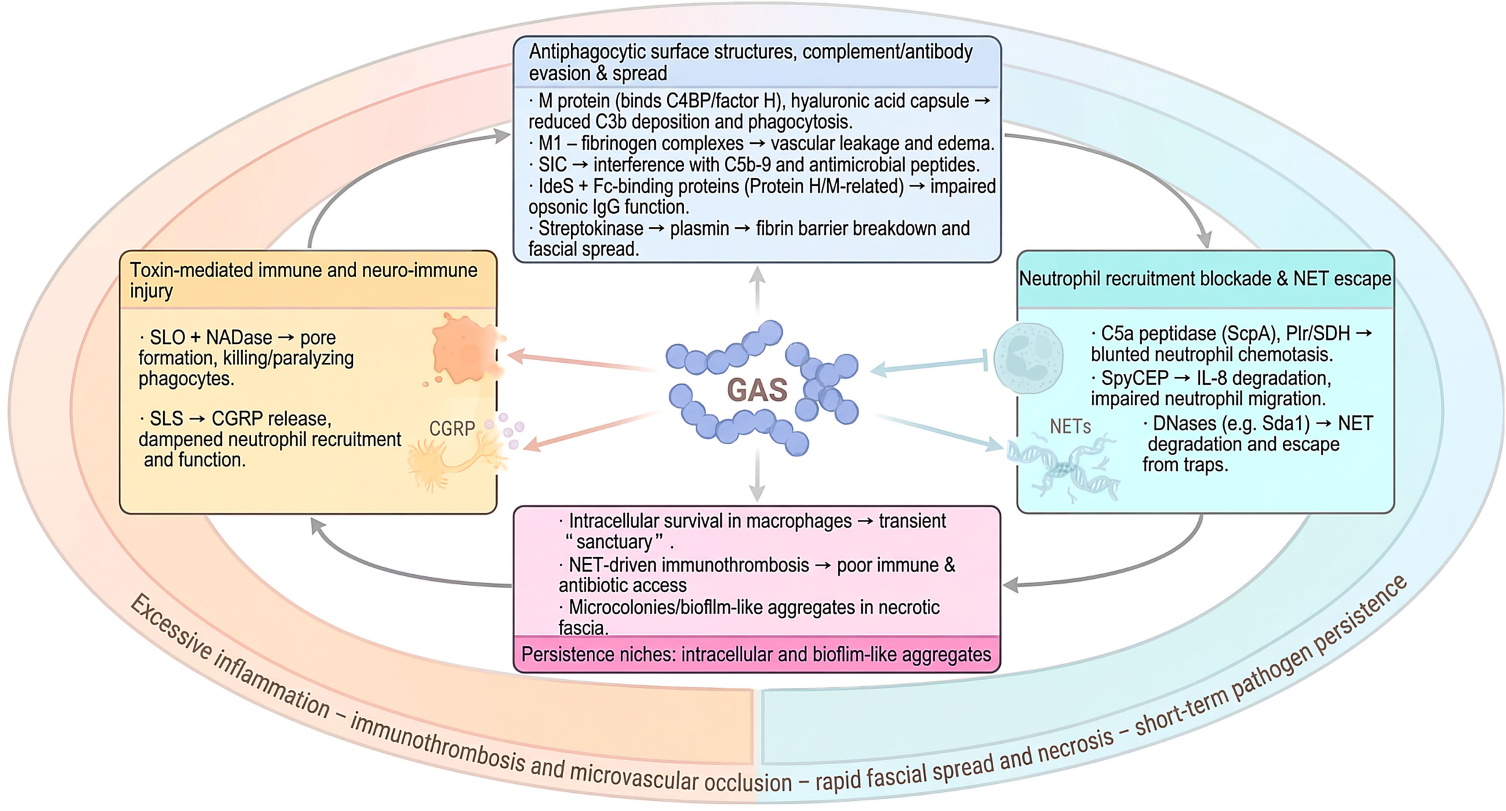
Multilayered immune-evasion and persistence strategies of Group A Streptococcus in necrotizing fasciitis. This schematic summarises the principal immune-evasion and persistence mechanisms of Group A Streptococcus (GAS) in NF. GAS reduces opsonophagocytic clearance through antiphagocytic surface structures and interference with complement and antibody function, impairs neutrophil recruitment and NET-mediated containment, and directly injures host immune cells through secreted toxins. In parallel, GAS promotes tissue spread and persistence through fibrinolysis, intracellular survival, and transient aggregate or biofilm-like niches within necrotic, hypoperfused tissue. Together, these mechanisms sustain bacterial burden, amplify tissue injury, and reinforce the need for urgent surgical source control.

Pathogen immune evasion also shapes the NF trajectory. Invasive pathogens can uncouple tissue injury from microbial clearance, allowing persistence in damaged, hypoperfused tissue and sustaining inflammation. Using GAS as an exemplar, this section summarizes antiphagocytic surface structures; complement and antibody interference; streptokinase-plasmin-mediated spread; proteases and DNases that impair neutrophil recruitment and NET containment; cytotoxins and neuro-immune interactions; and intracellular or aggregate-like persistence niches (**Figure 4**). These mechanisms can prolong tissue injury, sustain pathogen burden in damaged or hypoperfused tissue, and contribute to discordant local and systemic host-response patterns.

#### 2.9.1 Antiphagocytic and related evasive mechanisms

M protein-mediated antiphagocytosis. The GAS M protein family is central to evasion of complement-mediated opsonization. M1 strains can bind C4b-binding protein (C4BP), whereas other serotypes recruit factor H; both mechanisms reduce C3b deposition on the bacterial surface and thereby impair phagocytosis by neutrophils and macrophages (Ermert et al., 2013). M1 protein can also form complexes with fibrinogen, creating a physical barrier that masks opsonic sites. In addition, M1–fibrinogen complexes can trigger excessive activation of neutrophil β2-integrin Mac-1, leading to heparin-binding protein release, vascular leakage, edema, worsened tissue hypoperfusion, and reduced antimicrobial access (Herwald et al., 2004; Macheboeuf et al., 2011).

Hyaluronic acid capsule-mediated molecular mimicry. Many invasive GAS strains express a hyaluronic acid capsule that mimics host connective tissue components and reduces immune recognition. GAS mutants lacking capsule synthesis, such as those with hasA disruption, or naturally acapsular variants are more readily cleared by neutrophils and show attenuated virulence in experimental models (Solomon et al., 2018). The capsule therefore represents an important antiphagocytic structure that can support GAS persistence during invasive soft-tissue infection. Its contribution may vary by strain background and should not be assumed to apply uniformly across all β-hemolytic streptococci (Shimomura et al., 2011).

Interference with neutrophil recruitment and trafficking. GAS can also interfere with neutrophil recruitment. ScpA, a C5a peptidase, cleaves the potent chemoattractant C5a and blunts early neutrophil recruitment (Ji et al., 1996). SpyCEP, a CXCL8/IL-8 protease, degrades human CXCL8/IL-8 and thereby inhibits neutrophil transendothelial migration and weakens neutrophil-dependent containment; increased SpyCEP expression has been linked to invasive M1T1 lineages (Zinkernagel et al., 2008). In addition, the surface Plr/SDH protein can bind C5a and may further diminish chemotactic signaling (Terao et al., 2006). These mechanisms can spatially uncouple inflammatory tissue injury from efficient neutrophil-mediated bacterial clearance.

NET escape. GAS secretes multiple DNases, including Sda1, SdaD, and SpnA, that degrade the DNA backbone of neutrophil extracellular traps (NETs), enabling escape from NET structures and reducing NET-mediated killing. Phage-encoded Sda1 promotes dissemination and can dampen TLR9-dependent DNA sensing in mouse models; clinically, high DNase activity and the presence of sda1 have been associated with invasive phenotypes (Walker et al., 2007; Chang et al., 2011; Uchiyama et al., 2012).

Complement, antimicrobial peptide, and antibody interference. GAS counteracts additional host effector systems through specific virulence factors. The streptococcal inhibitor of complement (SIC) can interfere with membrane attack complex formation by binding nascent C5b-9 and limiting its insertion; it can also neutralize antimicrobial peptides, including LL-37 and α-defensins, thereby weakening neutrophil bactericidal activity (Fernie-King et al., 2004). GAS can further evade opsonic antibodies through at least two mechanisms: the cysteine protease IdeS cleaves IgG at the hinge region and disrupts Fcγ receptor-mediated phagocytosis, whereas surface proteins such as Protein H and M-related proteins can bind IgG Fc in an inverted orientation, impairing opsonophagocytic clearance (Wenig et al., 2004; Nordenfelt et al., 2012).

Protease-mediated remodeling by SpeB. SpeB is a secreted GAS cysteine protease with context-dependent effects on invasive disease. At the tissue level, SpeB can cleave host proteins and bacterial surface or secreted factors, thereby influencing matrix injury, immune recognition, and the availability of other virulence determinants. However, invasive M1T1 GAS can undergo regulatory or phenotypic shifts associated with reduced SpeB activity, which may preserve M protein, SLO, streptokinase, SIC, and other proteinaceous virulence factors from proteolytic degradation. Accordingly, SpeB should not be interpreted simply as a uniformly pro-necrotic protease; rather, its contribution to NF likely depends on strain background, regulatory state, infection niche, and the balance between local proteolysis and preservation of immune-evasion programs (Kansal et al., 2000; Aziz et al., 2004).

Activation of fibrinolysis to promote tissue spread. Streptokinase activates host plasminogen to plasmin, thereby promoting fibrin degradation and weakening fibrin-rich barriers that would otherwise help localize infection. In invasive soft-tissue infection, this pathway may facilitate bacterial escape from thromboinflammatory containment, promote spread along fascial planes, and interact with tissue hypoperfusion and necrosis to create permissive niches for pathogen persistence. Experimental studies have shown that human plasminogen can markedly increase susceptibility to GAS infection in a streptokinase-dependent manner, supporting streptokinase as a key determinant of host-species specificity and invasive potential (Sun et al., 2004). Disruption or inhibition of streptokinase-related pathways can limit dissemination or improve survival in model systems (Sun et al., 2012; Cook et al., 2014). Because streptokinase–plasminogen interactions are species-restricted, animal-model findings require careful interpretation, but this pathway remains an important example of how GAS manipulates host hemostasis to promote invasive disease.

Collectively, these evasive mechanisms reduce opsonization, impair neutrophil recruitment and NET-mediated containment, interfere with complement and antibody effector functions, neutralize antimicrobial peptides, and promote fibrin barrier breakdown. In damaged and hypoperfused tissue, such strategies can allow GAS to persist despite intense inflammation, thereby sustaining local tissue injury and potentially contributing to prolonged or dysregulated host-response trajectories. Rather than acting in isolation, these mechanisms likely interact with tissue ischemia, bacterial burden, source-control timing, and host susceptibility to shape the balance between pathogen clearance, inflammatory injury, and local-systemic immune dysregulation.

#### 2.9.2 Toxin-mediated immune-cell damage

GAS not only evades host defenses but can also directly subvert immune effector cells through secreted toxins, thereby impairing opsonophagocytic clearance even within a hyperinflammatory milieu. Key toxins involved in this process include streptolysin O (SLO), streptolysin S (SLS), and streptococcal NAD-glycohydrolase (NADase).

SLO-NADase cooperative intracellular injury. SLO is a pore-forming cytotoxin that injures leukocytes and platelets and can perforate phagosomal membranes, enabling co-secreted NADase to enter the host cytosol (Timmer et al., 2009; Bastiat-Sempe et al., 2014). NADase depletes NAD+, disrupts antimicrobial programs, and can impair phagolysosomal maturation, favoring intracellular GAS persistence (Bastiat-Sempe et al., 2014; O'Neill et al., 2016). Viable GAS has been reported within macrophages in NF tissue (Thulin et al., 2006), and sublytic SLO impairs neutrophil oxidative burst and killing before cell death (Uchiyama et al., 2015).

SLS-mediated cytotoxicity, hemolysis, and neuro-immune modulation. SLS is a ribosomally synthesized peptide toxin encoded by the sag operon and is a major determinant of the β-hemolytic phenotype of GAS. Beyond direct leukocyte injury, SLS can damage epithelial and soft-tissue cells, amplify inflammatory signaling, and contribute to necrotic lesion formation in experimental skin and soft-tissue infection models (Higashi et al., 2016). SLS-mediated erythrocyte lysis has been linked to disruption of the erythrocyte band 3 anion exchanger, leading to osmotic injury and hemolysis; this process may increase local availability of hemoglobin- and heme-associated iron, although the quantitative importance of this mechanism in human NF remains incompletely defined (Higashi et al., 2016). In parallel, experimental work suggests that SLS can activate nociceptive neurons and trigger release of calcitonin gene-related peptide (CGRP), which suppresses neutrophil recruitment and opsonophagocytic killing. In an NF mouse model, blockade of nociceptive signaling or CGRP antagonism improved bacterial clearance and reduced tissue pathology (Pinho-Ribeiro et al., 2018). Together, these findings support a model in which SLS contributes to NF-like disease through combined cytolytic, hemolytic, inflammatory, and neuro-immune mechanisms, while the relative contribution of each pathway in human NF likely varies by strain background, tissue niche, and host context.

In summary, SLO, NADase, and SLS can act together to injure immune effector cells, suppress key bactericidal functions at sublytic concentrations, promote intracellular persistence, and modulate neuro-immune control of local inflammation. By sustaining bacterial burden in damaged or ischemic tissues while amplifying local injury, these toxin-mediated mechanisms may interact with tissue hypoperfusion, source-control timing, pathogen burden, and host susceptibility to shape compartment-specific host-response patterns. In this setting, local inflammatory injury, impaired microbial clearance, and systemic immune dysfunction may coexist to varying degrees.

#### 2.9.3 Persistence versus clearance: intracellular niches and biofilm-like aggregation

NF is an acute invasive infection rather than a canonical chronic biofilm disease. Nonetheless, necrotic and hypoperfused tissue microenvironments, and in some settings foreign material, can create transient spatial niches that favor pathogen persistence and immune evasion.

Intracellular persistence. As discussed above, SLO-mediated membrane injury can facilitate delivery of NADase into host cells, disrupting antimicrobial cellular programs and promoting intracellular persistence of GAS within phagocytes, and in some settings limited intracellular replication (Timmer et al., 2009; Bastiat-Sempe et al., 2014; O'Neill et al., 2016). Pathological observations of intracellular GAS in patient tissues support the possibility of intracellular niches with reduced exposure to extracellular immune effectors and potentially altered antimicrobial susceptibility (Thulin et al., 2006). These findings do not imply that NF is primarily an intracellular infection, but they suggest that intracellular persistence may contribute to incomplete bacterial clearance in selected contexts.

Microcolony and biofilm-like aggregation. In necrotic tissue or on foreign surfaces, bacteria can form aggregates embedded within an extracellular matrix-rich milieu that may impede immune-cell access and reduce antimicrobial penetration. Although NF should not be regarded as a classic biofilm-driven chronic infection, indirect evidence from broader biofilm and chronic wound literature suggests that biofilm-like structures or microaggregates may arise transiently in devitalized or hypoperfused tissue environments (Roy et al., 2020; Yin et al., 2022). Such structures may help explain discrepancies between *in vitro* antimicrobial susceptibility and *in vivo* treatment response, but their frequency and functional importance in human NF remain incompletely defined.

Amplification by perfusion failure. Immunothrombosis and microcirculatory dysfunction can further limit immune-effector trafficking and antimicrobial delivery to infected fascia, thereby interacting with intracellular persistence and microbial aggregation. Surgical source control therefore remains pivotal: excision of devitalized tissue disrupts these niches, reduces bacterial burden, restores more favorable local conditions for immune and antimicrobial activity, and limits further tissue destruction (Anaya and Dellinger, 2007). Any host-directed immunomodulation must be interpreted within this context, because immune manipulation is unlikely to compensate for ongoing bacterial persistence in poorly perfused or inadequately debrided tissue.

Across Sections 2.3.1–2.3.3, GAS immune evasion can be organized into three interacting modules: antiphagocytic and innate-evasion programs that interfere with opsonization, complement activity, neutrophil trafficking, NET containment, and fibrin barriers; toxin-mediated immune-cell injury and neuro-immune modulation; and persistence niches formed by intracellular survival and transient microcolony or biofilm-like aggregation in necrotic, hypoperfused tissue (**Figure 4**). Together, these mechanisms can sustain bacterial burden despite intense inflammation, amplify local tissue injury, and contribute to prolonged or dysregulated host-response trajectories. Because these GAS-centered mechanisms are not necessarily recapitulated in polymicrobial NF, the next section contrasts GAS-predominant monomicrobial disease with polymicrobial disease to clarify etiology-linked immune heterogeneity and its implications for monitoring and adjunctive strategy selection.

## 3 GAS-predominant monomicrobial versus polymicrobial NF: immunopathological heterogeneity and clinical implications

Although NF can involve overlapping phases of early hyperinflammation and secondary immune dysfunction, the dominant immune drivers and clinical trajectories vary substantially by pathogen context. In particular, GAS-predominant monomicrobial disease and polymicrobial NF differ in microbial ecology, virulence programs, tissue microenvironment, inflammatory amplification, and tempo of systemic deterioration. Building on the mechanisms discussed above, this section compares the relative dominance of immune and pathophysiological drivers in GAS-predominant versus polymicrobial NF and considers how these differences may inform immune monitoring, source-control reassessment, and evidence-bounded adjunctive strategies. **Table 1** summarizes the major contrasts between GAS-predominant type II and polymicrobial type I NF.

**Table 1:** Key contrasts between GAS-predominant monomicrobial (type II) and polymicrobial (type I) necrotizing fasciitis.

Dimension	GAS-predominant monomicrobial NF (type II)	Polymicrobial NF (type I)
Typical setting/source	•Community-acquired	•Often in comorbid hosts and/or healthcare settings
	•Minor skin breach/trauma	•Perineal/abdominal sources (ulcers, post-op wounds, viscus perforation)
	•Extremities/trunk common	
Dominant pathobiology	•Toxin/superantigen-forward program + immune evasion	•Polymicrobial synergistic tissue injury (proteases/lipases) in devitalized tissue
	•Rapid systemic amplification	•hypoxia/acidosis prominent (± gas)
Immune activation profile	•Narrower PRR input	•Broader PAMP/PRR input
	•Toxin-coupled amplification often predominates	•More heterogeneous inflammatory pattern
Adaptive immune driver	•Superantigen-dominant T-cell activation → STSS	•Conventional antigen-specific/mixed cues more typical
		•toxin-like picture only in selected contexts (e.g., superantigen-producing S. aureus)
Clinical tempo/phenotype	•Abrupt onset	•Variable tempo
	•Early shock/MODS more common	•Severe sepsis phenotype typical
	•Toxic shock–like features more frequent	•Shock may evolve later
		•Classic toxin-mediated features less common
Biomarkers (exploratory)	•CXCL10/IP-10↑, IL-10↑	•MMP-9↑
		•IL-6 is a shared marker linked to septic shock across NSTI types
Adjunct priorities	•β-lactam + protein-synthesis inhibitor (clindamycin; linezolid alternative)	•Broad Gram-negative/anaerobe coverage (± MRSA)
	•Consider IVIG if STSS phenotype	•Repeat debridement often required
		•IVIG usually not supported
		•HBOT only if it does not delay surgery

### 3.1 Differences in virulence architecture

In GAS-predominant monomicrobial type II NF, virulence is often organized around a toxin- and superantigen-centered program layered onto coordinated immune-evasion mechanisms. As discussed above, these mechanisms include antiphagocytic surface structures such as M protein and the hyaluronic acid capsule; interference with neutrophil recruitment and NET-mediated containment through ScpA, SpyCEP, and DNases such as Sda1; protease-mediated remodeling by SpeB; complement and immunoglobulin interference mediated by factors such as SIC and IdeS; streptokinase–plasminogen-mediated fibrin barrier disruption; and cytotoxic injury driven by SLO, SLS, and NADase (Ji et al., 1996; Kansal et al., 2000; Aziz et al., 2004; Fernie-King et al., 2004; Herwald et al., 2004; Sun et al., 2004; Wenig et al., 2004; Terao et al., 2006; Thulin et al., 2006; Anaya and Dellinger, 2007; Walker et al., 2007; Zinkernagel et al., 2008; Timmer et al., 2009; Chang et al., 2011; Macheboeuf et al., 2011; Shimomura et al., 2011; Nordenfelt et al., 2012; Sun et al., 2012; Ermert et al., 2013; Bastiat-Sempe et al., 2014; Cook et al., 2014; Uchiyama et al., 2015; Higashi et al., 2016; O'Neill et al., 2016; Pinho-Ribeiro et al., 2018; Roy et al., 2020; Yin et al., 2022). Together, this architecture supports immune evasion, tissue invasion, and rapid systemic inflammatory amplification.

In contrast, polymicrobial type I NF typically reflects mixed aerobic–anaerobic microbial communities, often involving enteric Gram-negative bacilli and anaerobes within devitalized, hypoxic, or contaminated tissue environments. Pathogenicity in this setting is shaped less by a single dominant toxin–superantigen axis and more by interspecies synergy, cooperative tissue destruction, anaerobic metabolism, and local enzymatic injury, including protease- and lipase-mediated matrix disruption. The broader PAMP repertoire in polymicrobial disease may drive robust innate immune activation, but the inflammatory phenotype is generally more heterogeneous than in GAS-predominant disease. In selected cases, the presence of toxin-producing organisms, such as Staphylococcus aureus strains with superantigenic potential, may add an additional layer of inflammatory amplification (Spaulding et al., 2013).

### 3.2 Differences in innate immune activation patterns

At the level of early innate immune initiation, polymicrobial and GAS-predominant monomicrobial NF can exhibit distinct activation profiles because of differences in pathogen-derived signals and tissue context. Polymicrobial NF typically exposes the host to a broader repertoire of PAMPs derived from mixed aerobic–anaerobic communities, together with DAMPs released from devitalized tissue. This wider input spectrum may engage multiple PRR pathways and generate a heterogeneous inflammatory response. In contrast, GAS-predominant NF is often shaped by Gram-positive cell-wall sensing, TLR–MyD88-dependent priming, and toxin-coupled inflammasome activation, with streptolysin O providing a representative link between bacterial cytotoxicity and NLRP3-dependent IL-1 family cytokine maturation (Schwandner et al., 1999; Harder et al., 2009; Keestra-Gounder and Tsolis, 2017).

Consistent with these upstream differences, both etiologies can induce prominent neutrophil recruitment, NET formation, complement activation, and endothelial injury; however, the tempo and dominant amplification loops may differ. GAS-predominant disease more often presents with abrupt systemic toxicity, early circulatory compromise, and toxin-amplified inflammatory escalation, particularly when superantigen activity or STSS-like physiology is present. Polymicrobial NF may follow a more variable course, depending on the anatomical source, tissue perfusion, anaerobic burden, host comorbidities, and timing of source control. In such cases, inflammation may initially remain more localized before rapid deep-tissue extension and systemic deterioration occur (Wong et al., 2003; Saffarzadeh et al., 2012; Hakkarainen et al., 2014; Hansen et al., 2017; Rath et al., 2023).

### 3.3 Differences in adaptive immune involvement

At the level of adaptive immunity, a key distinction between GAS-predominant monomicrobial NF and typical polymicrobial NF is whether a superantigen axis dominates early systemic T-cell activation. In GAS-associated NF, superantigens can bypass conventional antigen-specific recognition and drive polyclonal T-cell activation, thereby contributing to abrupt systemic toxicity, early shock, multiorgan dysfunction, and STSS-like physiology (Lappin and Ferguson, 2009; Spaulding et al., 2013). This mechanism helps explain why GAS-predominant disease may present with a more explosive systemic inflammatory phenotype, sometimes accompanied by diffuse erythematous rash or other toxic shock features.

In typical polymicrobial NF, adaptive immune activation is more often shaped by conventional antigen-specific responses, broad innate inflammatory cues, tissue necrosis, and ongoing microbial burden rather than by a single dominant superantigen axis. Accordingly, polymicrobial disease more commonly follows a severe sepsis phenotype with a variable tempo of shock progression, influenced by pathogen burden, anatomical source, tissue perfusion, host comorbidities, and timing of source control. However, this distinction is not absolute: in selected polymicrobial contexts, the presence of superantigen-producing organisms such as Staphylococcus aureus may add toxin-mediated T-cell activation and contribute to a more toxic shock-like presentation (Spaulding et al., 2013).

### 3.4 Differences in compartmentalized hyperinflammatory and immunosuppressive patterns

Although both GAS-predominant and polymicrobial NF can involve overlapping hyperinflammatory and immunosuppressive programs, the timing, intensity, and dominant drivers of these trajectories may differ by etiology. GAS-associated NF often reaches an abrupt systemic inflammatory peak early in the disease course, particularly when toxin and superantigen activity contribute to shock-linked cytokine amplification (Hansen et al., 2017; Palma et al., 2021). Polymicrobial NF can likewise trigger severe systemic inflammation, but its inflammatory profile is usually less dominated by a single toxin–superantigen axis and may be more strongly shaped by tissue hypoxia, acidosis, microbial synergy, devitalized tissue burden, and delayed source control.

GAS-predominant NF may therefore be characterized by early toxin-amplified systemic inflammation while, in some patients, peripheral immune dysfunction emerges in parallel with ongoing wound inflammation, tissue hypoperfusion, or pathogen persistence. Polymicrobial NF may present a more spatially heterogeneous mixture of systemic inflammation, local hypoxia, necrosis-associated dysfunction, impaired microbial clearance, and counter-regulation. With ICU-level severity, different etiologies may share immunosuppressive signatures, including low mHLA-DR and impaired innate responsiveness, while retaining distinct local microbial and tissue-compartment drivers (van der Poll et al., 2017; Leijte et al., 2020).

### 3.5 Biomarker differences and subtyping potential

Early immune biomarker panels may refine etiologic and immunological subtyping, but remain exploratory. In a prospective NSTI cohort, a multianalyte signature including CXCL10/IP-10, IL-10, MMP-9, and other mediators distinguished type II monomicrobial from type I polymicrobial infection with moderate performance (AUC >0.7), with CXCL10/IP-10 strongest in validation (Palma et al., 2021). MMP-9 tended to be higher in type I, whereas CXCL10/IP-10 and IL-10 were higher in type II. IL-6 was a shared shock marker across NSTI types, reflecting severity rather than pathogen-specific biology (Palma et al., 2021).

### 3.6 Etiology-informed implications for monitoring and adjunctive care

For all forms of NF, the non-negotiable foundations of care remain prompt surgical source control, broad-spectrum empiric antimicrobial therapy with subsequent pathogen-directed refinement, and high-quality organ support. Etiologic differences should not delay these measures, but they may inform early priorities, adjunctive considerations, and interpretation of host-response trajectories.

In GAS-associated type II NF, particularly when STSS-like physiology is present, antimicrobial therapy should generally include a β-lactam combined with a protein-synthesis inhibitor, classically clindamycin, to reduce exotoxin and superantigen production. Linezolid may be considered in selected settings, including clindamycin resistance, intolerance, or the need for concurrent MRSA coverage (Stevens et al., 2014). In patients with severe early STSS-like physiology or rapidly progressive organ dysfunction, IVIG may be considered on an individualized basis; however, clinical efficacy remains uncertain, and its use should be interpreted in relation to timing, severity, antitoxin rationale, and risk–benefit assessment (Darenberg et al., 2003). The mechanistic basis for these antitoxin strategies derives from the superantigen and humoral neutralization pathways discussed above (Lappin and Ferguson, 2009; Spaulding et al., 2013).

In polymicrobial type I NF, early priorities center on reliable coverage of mixed aerobic–anaerobic flora, including enteric Gram-negative organisms and anaerobes, with MRSA coverage when clinically indicated, together with repeated reassessment of tissue viability and adequacy of debridement (Stevens et al., 2014). In this setting, IVIG is not routinely supported unless a clear toxin-mediated or STSS-like phenotype is suspected. Hyperbaric oxygen therapy may be considered as an adjunct in selected centers, particularly when logistical conditions allow, but the evidence base remains largely observational and it should never delay definitive surgical source control or ICU-level stabilization (Huang et al., 2023).

From a host-response perspective, etiology should be integrated with immune phase, tissue perfusion, and source-control status rather than used in isolation. Early GAS-predominant disease may justify greater attention to antitoxin strategies and rapid recognition of STSS-like physiology, whereas polymicrobial disease requires particular attention to anatomical source, anaerobic burden, devitalized tissue, and repeated debridement. Host-directed immunomodulation remains investigational: anti-inflammatory approaches should not compromise pathogen clearance during early hyperinflammation, and immune-restorative strategies should be reserved for future biomarker-stratified studies or rigorously monitored protocols in patients with documented immunoparalysis after adequate source control.

## 4 Host-related susceptibility and baseline immune reserve

NF outcomes reflect pathogen virulence, timing and adequacy of source control, and host factors that shape susceptibility, immune tone, perfusion, and repair. Key determinants include immunogenetic variation, epigenetic and transcriptional priming, immunometabolic constraints, and barrier, tissue, and microvascular integrity. These factors may bias the early course toward toxin-amplified hyperinflammation, inadequate containment, impaired repair, or earlier immune dysfunction, and may help prioritize future risk stratification and immune monitoring.

### 4.1 Genetic background

Genetic variation can shape both susceptibility to NF and the magnitude, quality, and timing of downstream immune responses. In GAS-associated NF and STSS, HLA class II polymorphisms are particularly relevant because they modulate superantigen–MHC class II interactions and the extent of downstream polyclonal T-cell activation. For example, HLA-DQA1*01:03 has been associated with an increased risk of invasive GAS disease, whereas the HLA-DRB1*15:01–DQB1*06:02 haplotype has been reported as relatively protective in STSS cohorts (Llewelyn, 2005; Parks et al., 2020). These associations support the concept that host immunogenetic background can influence the intensity of toxin-driven systemic inflammation.

Beyond HLA, innate immune signaling variants may influence NF, although direct evidence is limited. A truncating NFKB1 variant has been linked to recurrent NSTI with excessive NLRP3 inflammasome activation and type I interferon signaling (Nurmi et al., 2024). Inflammasome-activating variants, including gain-of-function NLRP3 or altered purinergic signaling, are biologically plausible contributors to IL-1-skewed hyperinflammation (Menu and Vince, 2011). TLR4 Asp299Gly/Thr399Ile and CTLA-4-related pathways have been associated with sepsis susceptibility or outcomes, but NF validation is lacking (Walker, 2013; Mu et al., 2024).

Overall, aside from HLA-associated modulation of GAS superantigen responses and rare illustrative variants such as NFKB1, most proposed genetic links remain insufficiently validated in NF. Adequately powered immunogenomic studies in well-phenotyped NF cohorts are therefore needed to define how host genotype interacts with pathogen context, tissue perfusion, and source-control timing. Collectively, host genetic background may influence the threshold for toxin-amplified inflammation, the propensity toward immune dysfunction, and the pace of recovery, helping to explain why patients with similar pathogen exposures can follow divergent clinical trajectories.

### 4.2 Epigenetics and trained immunity

Epigenetic and immunometabolic reprogramming may shape host-response trajectories in NF by altering chromatin accessibility, transcriptional responsiveness, and functional set points in innate immune cells. Although direct NF-specific evidence remains limited, findings from severe infection and sepsis biology suggest that monocytes and macrophages can acquire durable transcriptional states after intense inflammatory exposure. In severe infection, sustained chromatin remodeling and DNA methylation-associated silencing can promote an endotoxin tolerance-like phenotype, characterized by blunted induction of proinflammatory cytokines, such as TNF-α and IL-6, upon restimulation (El Gazzar et al., 2008; Cross et al., 2019).

In contrast, trained immunity, best described after exposures such as β-glucan, is mediated by coordinated histone modifications, including H3K4me3 and H3K27ac, together with metabolic rewiring that primes myeloid cells for enhanced responses upon rechallenge (Moorlag et al., 2020; Netea et al., 2020). Cytokine priming can also generate memory-like NK-cell programs with heightened IFN-γ production (Romee et al., 2012). These adaptive features of innate immune cells illustrate how prior exposures, inflammatory history, and metabolic context may bias the subsequent response to invasive infection toward either amplified inflammation or impaired responsiveness.

For NF, the relevance of these mechanisms is likely greatest in two settings: first, during the early phase, when pre-existing trained or primed innate programs may lower the threshold for toxin- or PRR-driven hyperinflammation; and second, during recovery, when persistent tolerance-like transcriptional programs may contribute to immunoparalysis, secondary infection risk, and delayed tissue repair. Prolonged transcriptional changes documented in sepsis survivors support the plausibility of persistent immune reprogramming after critical illness, although whether similar programs occur in NF survivors requires direct investigation (Laudanski et al., 2021). Epigenetic-targeted approaches, such as modulation of histone acetylation or DNA methylation, show preclinical potential in sepsis-associated immune dysfunction but remain investigational and unevaluated in NF-specific clinical contexts (Cross et al., 2019).

### 4.3 Impact of metabolism and chronic disease

Obesity and diabetes can shape NF susceptibility through immunometabolic baseline, perfusion, and repair capacity. Obesity is associated with chronic low-grade inflammation and myeloid reprogramming that may potentiate infectious triggers. Western diet- or obesity-associated signals can induce NLRP3-dependent trained immunity-like programs (Christ et al., 2018). Metabolic by-products may also intersect with innate sensing; NOD1 ligands have been linked to inflammatory signaling and metabolic inflammation (Schertzer et al., 2011).

In contrast, diabetes-associated hyperglycemia, advanced glycation end-products, and microvascular dysfunction can impair neutrophil chemotaxis, phagocytosis, oxidative killing, and coordinated tissue repair (Thimmappa et al., 2023). These defects may compromise early pathogen containment, favor deeper tissue extension, and delay wound-bed recovery after debridement. In addition, immunosuppressive exposures, such as systemic glucocorticoids, can further lower baseline antimicrobial competence and may accelerate the emergence of secondary immune dysfunction during critical illness. Clinically, these host backgrounds may bias NF toward divergent trajectories: amplified early hyperinflammation on a primed inflammatory baseline in some patients, or inadequate early pathogen control with rapid spread, tissue necrosis, and earlier dominance of immune dysfunction in others.

Direct evidence linking these immunometabolic mechanisms to NF-specific outcomes remains limited. Nevertheless, they provide a biologically plausible framework for understanding host heterogeneity in severe soft-tissue infection and support the integration of metabolic status, glycemic control, nutritional reserve, tissue perfusion, and immune-monitoring priorities into future NF risk-stratification studies.

### 4.4 Skin barrier integrity and microbiome

An intact cutaneous barrier and balanced skin microbiota form a first defense against invasive soft-tissue infection. Commensals provide colonization resistance and shape local immune tone through antimicrobial activity, pathogen competition, and host-microbe signaling (Naik et al., 2015; Chen et al., 2018). Trauma, dermatoses, tinea pedis, fissuring, ulcers, or surgical wounds create portals of entry. Environmental exposure can shift likely pathogens, including Vibrio vulnificus after seawater or raw seafood exposure and Aeromonas species after freshwater exposure.

Barrier- and microbiome-oriented measures are currently most relevant to prevention and post-acute risk reduction rather than acute immunomodulation. These include optimization of skin integrity, treatment of tinea pedis or chronic fissuring, careful wound care, and, in selected surgical contexts, preoperative decolonization strategies for Staphylococcus aureus (Tang et al., 2020). Interventions that deliberately manipulate commensal organisms, including topical probiotics or live biotherapeutic approaches, remain exploratory and should be viewed as future preventive or recovery-oriented strategies rather than established interventions for acute NF (Chen et al., 2018).

Taken together, host genetics, epigenetic and transcriptional imprinting, metabolic context, tissue perfusion, and the skin–microbiome–barrier interface collectively define the immunological “starting line” for NF. Better characterization of these determinants may improve early risk stratification, help prioritize immune-monitoring strategies, and support more individualized assessment of infection risk, tissue viability, wound healing, and recovery across the host-response trajectory.

## 5 Immune monitoring and trajectory-based assessment in critical care

Effective management of NF requires not only rapid surgical source control and antimicrobial therapy but also serial assessment of host immune function, inflammatory burden, and tissue perfusion. Such trajectory-based assessment refers to longitudinal, data-informed phenotyping that integrates immune monitoring with evaluation of regional ischemia, microvascular dysfunction, and wound-bed viability. The goal is not to replace clinical judgment or urgent operative reassessment, but to support time-sensitive decisions regarding repeat debridement, antimicrobial adequacy, organ support, secondary infection surveillance, and, in selected investigational or protocolized settings, phase-appropriate adjunctive strategies.

Bedside examination and routine laboratory testing remain foundational. However, a growing toolkit—including circulating biomarker panels, cellular immunophenotyping, functional immune assays, and imaging approaches that assess regional perfusion or inflammatory activity—may improve recognition of host-response heterogeneity and refine risk stratification. Because local and systemic immune states may be discordant, peripheral immune markers should not be interpreted as direct surrogates for the wound or fascial microenvironment. Instead, they should be integrated with bedside examination, wound evolution, operative findings, microbiology, and tissue-perfusion assessment. In this section, we review practical and emerging approaches for monitoring immune and perfusion trajectories in NF, emphasizing their potential utility, evidence boundaries, and limitations.

### 5.1 Bedside indicators and conventional risk tools

The Laboratory Risk Indicator for Necrotizing Fasciitis (LRINEC) uses C-reactive protein, white blood cells, hemoglobin, sodium, creatinine, and glucose to help distinguish NF from other severe soft-tissue infections. Its performance is variable, especially at thresholds such as ≥6, and sensitivity is insufficient to exclude early NF (Chang et al., 2019). Scores may be nondiagnostic before marked biochemical derangement. LRINEC also correlates imperfectly with shock, mortality, and cytokine responses (Hansen et al., 2017); it is an adjunctive risk tool and must not delay exploration when suspicion is high.

Among conventional markers of systemic stress and tissue hypoperfusion, serum lactate is one of the most consistent prognostic indicators. Markedly elevated lactate at presentation, often above 5–6 mmol/L in high-risk NSTI cohorts, has been associated with increased risk of shock and mortality (Yaghoubian et al., 2007; Chang et al., 2019). Serum creatinine provides additional information on renal dysfunction, systemic hypoperfusion, and evolving multiorgan injury. The neutrophil-to-lymphocyte ratio (NLR) offers a readily available composite marker of innate inflammatory activation and relative lymphopenia and has been associated with disease severity and mortality in observational NF cohorts (Wu et al., 2023). CRP and procalcitonin may help characterize inflammatory burden and bacterial infection severity, although their values are nonspecific and should be interpreted in relation to the clinical trajectory.

Although cytokines such as IL-6 and IL-10 may provide more direct insight into inflammatory and counter-regulatory immune states, routine use is limited by assay availability, turnaround time, and lack of NF-specific decision thresholds. In practice, bedside assessment should therefore emphasize serial trends in readily available indicators, including lactate clearance, creatinine trajectory, CRP or procalcitonin kinetics, leukocyte and lymphocyte counts, NLR, vasopressor requirements, organ dysfunction scores, and wound evolution. These conventional measures cannot define immune phase on their own, but they provide an essential clinical scaffold for deciding when more advanced immune monitoring or perfusion-focused reassessment may be warranted.

### 5.2 Immune functional phenotypes

Building on the mechanistic framework of immunoparalysis-like host-response states outlined in Section 2.2, functional immune monitoring may help translate these biological processes into clinically interpretable trajectories. Here, mHLA-DR expression, lymphocyte indices, cytokine patterns, and multiparametric immune profiles are considered from a monitoring perspective, with emphasis on clinical interpretation, evidence boundaries, and integration with source-control assessment. Serial mHLA-DR measurement may provide a practical readout of antigen-presenting capacity, whereas persistent lymphopenia may indicate impaired adaptive immune recovery; however, both signals should be interpreted alongside infection trajectory, source-control status, antimicrobial response, organ dysfunction, wound evolution, and ICU exposures (Hotchkiss et al., 2013; van der Poll et al., 2017; Leijte et al., 2020).

At present, NF-specific thresholds for mHLA-DR expression, lymphocyte subsets, cytokine panels, or ex vivo stimulation assays remain undefined, and their feasibility in routine practice varies substantially. Serial lymphocyte counts and NLR are widely available and can be trended rapidly, but they provide only indirect information about immune function. By contrast, mHLA-DR measurement may offer a more functional readout of antigen-presenting capacity, yet it usually requires flow-cytometry capability, preanalytical standardization, laboratory expertise, and locally validated reporting procedures. Inter-laboratory variability, sample handling, gating strategies, and turnaround time may limit its immediate applicability in urgent debridement or early antimicrobial decisions. Similarly, cytokine panels and ex vivo stimulation assays may provide mechanistic or prognostic information, but they are often unavailable in routine clinical workflows, may return too slowly for time-critical decisions, and lack validated NF-specific action thresholds.

Therefore, these tools should not be used as stand-alone diagnostic or therapeutic triggers. Their current value lies in supporting longitudinal risk stratification, secondary-infection surveillance, recognition of persistent immune dysfunction, and enrichment of future biomarker-guided studies rather than replacing bedside assessment or operative judgment. Emerging NSTI biomarker panels, including combinations involving IL-6, G-CSF, S100A8, CXCL10/IP-10, IL-10, and MMP-9, may further support etiologic or severity stratification, but they remain exploratory and require prospective validation, assay standardization, and workflow integration before routine clinical implementation (Palma et al., 2021).

### 5.3 Dynamic trajectories and outcome prediction

Once treatment has been initiated, longitudinal trends are likely to be more informative than isolated measurements. Concordant improvement in clinical status, wound appearance, vasopressor requirement, organ dysfunction, lactate clearance, and inflammatory-marker kinetics is generally reassuring. Conversely, discordant or biphasic trajectories, such as persistent shock despite debridement, recurrent fever, rising CRP or procalcitonin after an initial decline, worsening organ dysfunction, failure of lymphocyte recovery, or persistently depressed mHLA-DR where available, should prompt reassessment for inadequate source control, residual necrotic tissue, antimicrobial mismatch, secondary infection, or noninfectious inflammatory complications (Yaghoubian et al., 2007; Chang et al., 2019; Leijte et al., 2020; Wu et al., 2023).

Because validated NF-specific intervention thresholds are lacking, trajectory-based assessment should remain supportive rather than prescriptive. Discordance between local and systemic signals should be interpreted clinically rather than forced into a single immune phase. For example, improving circulating inflammatory markers or partial recovery of peripheral immune indices does not exclude residual necrotic tissue, persistent pathogen burden, or regional hypoperfusion; conversely, persistent lymphopenia or depressed mHLA-DR does not necessarily imply resolution of local wound inflammation. Immune-monitoring signals should therefore be integrated with bedside examination, wound evolution, microbiology, imaging, operative findings, and tissue-perfusion assessment before advanced immune monitoring or protocolized adjunctive evaluation is pursued. This approach preserves the clinical primacy of repeated source-control assessment while allowing immune and inflammatory trajectories to contribute to risk stratification and future trial-ready phenotyping.

### 5.4 Imaging and perfusion assessment for source-control decisions

In addition to laboratory and immune-monitoring parameters, imaging can provide spatial information on disease extent and, in selected settings, functional readouts relevant to tissue perfusion and viability. These techniques are adjunctive: they should not replace prompt surgical exploration when NF is strongly suspected, but they may help define fascial involvement, identify perfusion compromise, guide operative planning, and support decisions regarding re-exploration.

Conventional imaging mainly supports anatomical definition of disease extent, whereas selected intraoperative or functional methods may add information about perfusion and tissue viability. MRI can show deep fascial T2/STIR hyperintensity, while absent or reduced enhancement may indicate hypoperfusion or nonviable fascia (Kwee and Kwee, 2022; Spinnato et al., 2022; Wei et al., 2024). CT rapidly depicts gas, fascial fluid, and fascial-plane separation, offering actionable anatomic evidence (Kwee and Kwee, 2022; Spinnato et al., 2022). POCUS can detect fascial thickening, fluid tracking, and gas, but is operator-dependent and cannot exclude NF when negative (Castleberg et al., 2014; Gan et al., 2023). Intraoperative ICG angiography provides real-time perfusion assessment and may help define debridement margins, although validation remains limited (Ray et al., 2024).

Functional and molecular imaging may assess inflammatory activity, leukocyte trafficking, thrombosis biology, or oxygenation, but remains limited in NF. Labeled leukocyte scintigraphy can identify residual inflammatory foci but lacks emergency temporal and spatial resolution (Palestro, 2016; Censullo and Vijayan, 2017). FDG PET/CT may help selected difficult cases but has limited NF evidence and logistical barriers (Baberwal et al., 2024). Emerging neutrophil elastase, fibrin, and oxygenation approaches are largely preclinical or investigational and not NF-specific (Uppal et al., 2011; Li et al., 2018; Puuvuori et al., 2023; Hu et al., 2025).

Histopathological examination can provide confirmatory evidence by demonstrating features such as neutrophil-rich fasciitis, microvascular thrombosis, and tissue necrosis. However, a negative frozen section does not exclude early NF and should not delay surgery when clinical suspicion remains high (Solomon et al., 2018). Overall, imaging, histopathology, and functional assessment modalities should be regarded as adjuncts to clinical judgment and emergent surgical decision-making. Their core value lies in supporting assessment of disease extent, tissue perfusion, viability, and the need for re-exploration. Future integration of localized imaging readouts with systemic immune monitoring may enable more precise risk stratification and trial-ready phenotyping in NF.

## 6 Phase-informed adjunctive strategies and evidence boundaries

### 6.1 Foundational principles of NF management

NF management is a time-critical surgical emergency anchored by immediate source control, prompt effective antimicrobials with toxin suppression when GAS is suspected or confirmed, and critical care support. Debridement should occur as early as possible and be repeated as needed to remove devitalized or poorly perfused tissue. No laboratory score, biomarker, or imaging test should delay exploration when suspicion is high. Persistent shock, rising markers, worsening organ dysfunction, or progressive necrosis should prompt re-exploration (Stevens et al., 2014).

Empiric antimicrobial therapy should provide broad coverage for GAS, Staphylococcus aureus, including MRSA when relevant, Gram-negative bacilli, and anaerobes, followed by de-escalation according to microbiology, susceptibility testing, operative findings, and clinical trajectory. When GAS infection is confirmed or strongly suspected, high-dose β-lactam therapy combined with a protein-synthesis inhibitor, classically clindamycin, is recommended to suppress exotoxin and superantigen production and mitigate toxin-driven systemic effects (Zimbelman et al., 1999; Stevens et al., 2014). Where clindamycin cannot be relied upon because of resistance, intolerance, or local epidemiology, alternative toxin-suppressive strategies, including linezolid in selected contexts, should be considered according to microbiology, severity, and the need for concurrent MRSA activity (Chen et al., 2011; Stevens et al., 2014).

Critical care support should proceed in parallel with surgical and antimicrobial management, following established principles for sepsis and critical illness. Key priorities include early hemodynamic resuscitation, timely vasopressor support when indicated, organ support such as mechanical ventilation or renal replacement therapy, avoidance of both severe hyperglycemia and hypoglycemia, and early nutrition support when feasible. Importantly, surgical source control should be treated as a primary component of resuscitation rather than a delayed procedure after medical stabilization (Stevens et al., 2014).

These foundational interventions establish the conditions under which adjunctive strategies can be interpreted safely. A phase-informed approach does not replace surgery, antimicrobial therapy, or organ support; rather, it helps frame when the dominant problem is toxin-amplified hyperinflammation, persistent tissue hypoperfusion and source-control failure, or evolving immune dysfunction. Accordingly, all host-directed adjuncts discussed below should be considered evidence-bounded, phenotype-dependent, and subordinate to adequate source control and infection control.

### 6.2 Phase-adapted host-directed strategies: support, antitoxin therapy, and immune restoration

Host-directed adjuncts in NF should be conceptualized within an evidence-bounded, phenotype-informed framework that remains subordinate to urgent source control, effective antimicrobial therapy, and high-quality critical care. The terms Phase I and Phase II are useful for organizing dominant host-response patterns, but they should not be used as rigid treatment stages. In practice, toxin-amplified systemic inflammation, persistent wound-bed inflammation, tissue hypoperfusion, ongoing pathogen burden, and peripheral immune dysfunction may coexist in the same patient. Therefore, adjunctive strategies should be selected according to the dominant clinical problem, pathogen context, source-control status, tissue perfusion, and available immune-monitoring data rather than by chronological phase alone.

**Figure 5:**
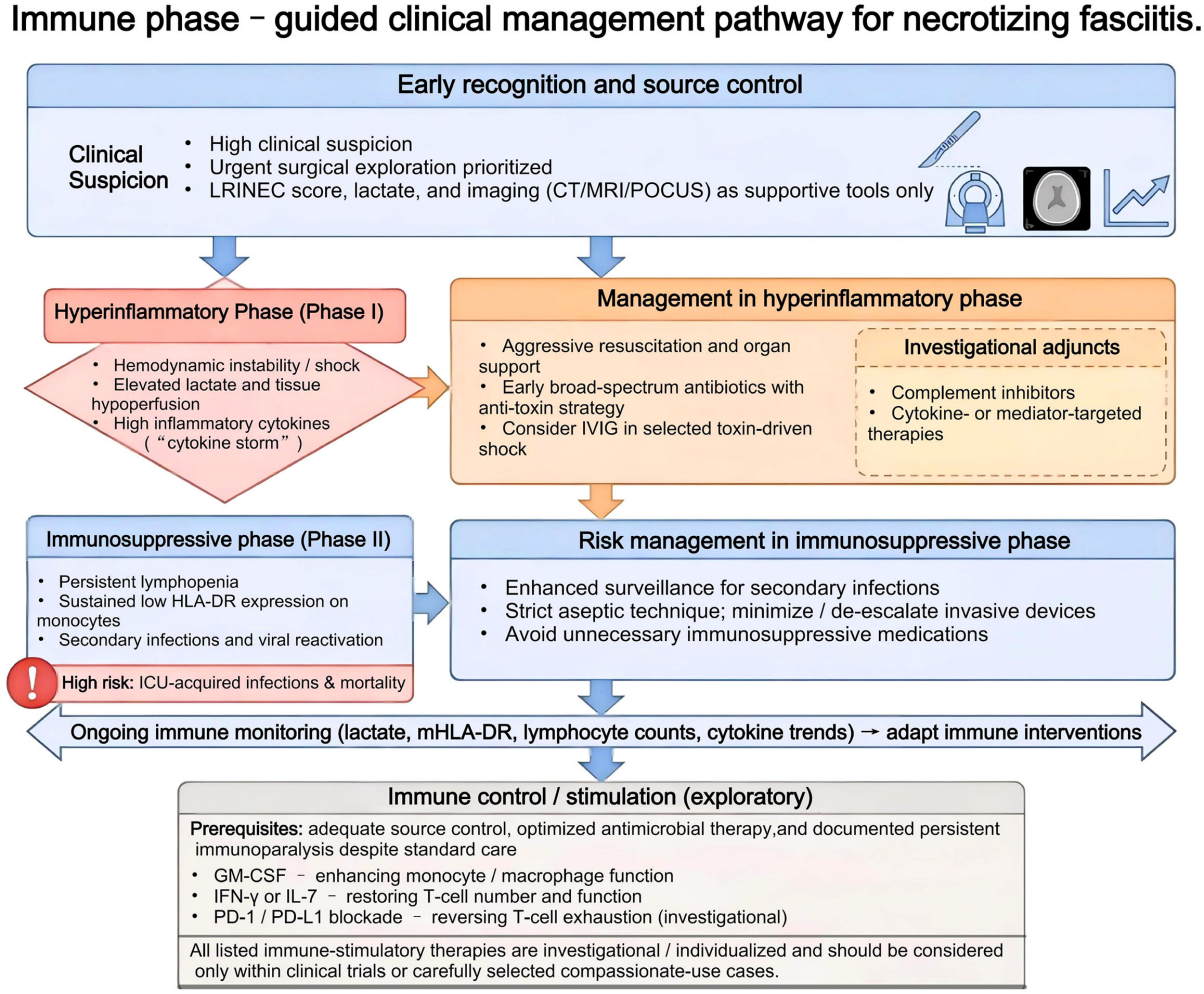
Phenotype-informed adjunctive framework for necrotizing fasciitis. The image integrates early recognition, urgent source control, and immune phase-aware adjunctive management in NF. Initial priorities are high clinical suspicion, prompt surgical exploration, broad-spectrum antimicrobial therapy, and haemodynamic and organ support; laboratory scores and imaging are supportive only and should not delay surgery. During the early hyperinflammatory phase, adjunctive priorities may include toxin-suppressive antimicrobial strategies and, in selected toxin-driven presentations, intravenous immunoglobulin, whereas other anti-inflammatory approaches remain investigational. During the later immunosuppressive phase, emphasis shifts to serial immune monitoring, prevention and surveillance of secondary infection, minimisation of unnecessary iatrogenic immunosuppression, and, in highly selected patients with persistent immunoparalysis after adequate source control, consideration of investigational immune-restorative strategies.

Antitoxin strategies may be considered in fulminant toxin-driven disease, especially GAS NF with STSS-like physiology. Selectively anti-inflammatory approaches remain investigational and require phenotype selection, timing, and safety criteria. Immune-restorative strategies should be evaluated only when objective immune dysfunction is documented and after residual infection, inadequate source control, and uncontrolled inflammatory escalation have been repeatedly reassessed. Given the limited NF-specific evidence, immune adjuncts should preferably remain within biomarker-guided trials, approved protocols, or rigorously monitored investigational settings (Hotchkiss et al., 2013). **Figure 5** summarizes this phenotype-informed clinical framework, integrating urgent source control with cautious adjunctive considerations across hyperinflammatory, hypoperfused, and immunosuppressive response patterns.

#### 6.2.1 Phase I: source control, antitoxin therapy, and evidence-bounded anti-inflammatory adjuncts

(i) During Phase I, priorities are source control, antimicrobials, and organ support in marked hyperinflammation or toxin-driven disease. Adjunctive immunomodulation must never delay debridement or antibiotics. Phase I adjuncts include antitoxin antibiotics, selective IVIG for STSS-like phenotypes, and investigational anti-inflammatory or complement-directed approaches.

(ii) Antitoxin antibiotics. In toxin-driven GAS NF, clindamycin suppresses exotoxin and superantigen production and may retain value in high-inoculum or stationary-phase states. Guidelines recommend a beta-lactam backbone, classically high-dose penicillin, plus clindamycin in GAS NF, especially with STSS physiology (Zimbelman et al., 1999; Stevens et al., 2014). Observational data support clindamycin in invasive GAS syndromes (Zimbelman et al., 1999; Stevens et al., 2014). Because resistance varies, susceptibility should be reviewed. Linezolid may be considered when MRSA coverage is needed or clindamycin cannot be used, guided by microbiology, severity, and local susceptibility (Chen et al., 2011; Stevens et al., 2014). For suspected toxin-mediated Staphylococcus aureus, inducible clindamycin resistance should be assessed when clindamycin is contemplated (Stevens et al., 2014).

(iii) IVIG for selected STSS-like phenotypes. IVIG is a selective antitoxin adjunct for severe GAS NF with STSS features. Mechanisms include superantigen neutralization, enhanced opsonophagocytosis, and immunomodulation. A small STSS randomized trial suggested possible benefit but was underpowered; observational studies and meta-analyses report lower mortality associations in clindamycin-treated STSS cohorts (Darenberg et al., 2003; Parks et al., 2018). In broader NSTI/NF, evidence is limited, and the INSTINCT trial did not improve the primary endpoint overall (Madsen et al., 2017; Bergsten et al., 2020). IVIG is therefore best reserved for individualized consideration in severe GAS/STSS-like shock or rapid organ dysfunction; routine use in polymicrobial NF is not supported.

(iv) Investigational anti-inflammatory and complement-directed approaches. These therapies remain investigational in NF. Broad cytokine antagonism has not produced consistent sepsis benefit, and anti-TNF strategies are not recommended (Hotchkiss et al., 2016; Cao et al., 2023). IL-1 blockade with anakinra has shown signals in sepsis patients with macrophage activation syndrome-like features, supporting biomarker-informed selection (Shakoory et al., 2016). Candidate hyperinflammatory signatures may include high IL-6, IL-1-skewed patterns, ferritin/IL-18 elevation, or MAS-like features, but these are selection frameworks, not indications (Leventogiannis et al., 2022). TREM-1 and complement-directed strategies are being explored, but NF evidence is lacking and use should be limited to trials or rigorously monitored investigational settings after source control (Siskind et al., 2022).

#### 6.2.2 Phase II: secondary immune dysfunction, infection surveillance, and investigational immune restoration

After initial source control has been achieved or is actively underway, and uncontrolled infection is being repeatedly reassessed, management priorities may expand toward detecting persistent immune dysfunction, preventing healthcare-associated or opportunistic infection, and supporting recovery. This phase should not be interpreted as a routine indication for immune stimulation. Rather, immune-restorative strategies should be considered only in investigational or protocolized settings when objective evidence of immune dysfunction is present, such as sustained lymphopenia, persistently depressed mHLA-DR, impaired ex vivo leukocyte responsiveness, or recurrent secondary infection despite adequate source control (Boomer et al., 2011; Guignant et al., 2011; Hotchkiss et al., 2013; van der Poll et al., 2017; Leijte et al., 2020).

Cytokine-based immune stimulation represents one investigational approach. GM-CSF and IFN-γ aim to restore monocyte/macrophage activation and antigen-presenting capacity, whereas IL-7 supports T-cell survival and lymphocyte recovery. In sepsis studies enriched for immune dysfunction, GM-CSF increased mHLA-DR expression, IFN-γ restored monocyte activation, and IL-7 increased T-cell numbers with acceptable early safety signals (Docke et al., 1997; Meisel et al., 2009; Francois et al., 2018). However, clinical outcome benefit remains unproven, NF-specific data are lacking, and mistimed stimulation during persistent infection or unresolved hyperinflammation could be harmful. These agents should therefore be evaluated only in biomarker-defined patients after adequate source control and with predefined safety criteria.

Checkpoint-based immune restoration is another exploratory strategy for patients with exhaustion-like T-cell dysfunction. PD-1/PD-L1 upregulation has been associated with impaired T-cell function and secondary infection risk in critical illness (Guignant et al., 2011; Hotchkiss et al., 2013; van der Poll et al., 2017). Preclinical sepsis models suggest that PD-1/PD-L1 modulation can restore immune function in context-dependent ways, and an early-phase septic-patient trial found single-dose nivolumab to be generally tolerated, with pharmacodynamic immune-restoration signals in selected cohorts (Hotchkiss et al., 2019). Nevertheless, clinical benefit and NF efficacy remain unproven, and checkpoint inhibitors should remain confined to clinical trials or rigorously monitored investigational protocols (Guignant et al., 2011; Hotchkiss et al., 2013; Xiang et al., 2025).

Safety boundaries are central to any Phase II immune-restorative approach. Persistent shock, renewed inflammatory escalation, worsening organ dysfunction, residual necrotic tissue, antimicrobial mismatch, or suspected secondary infection should trigger source-control and infection reassessment before immune-stimulating therapy is initiated, continued, or escalated. Serial immune monitoring may support trial enrichment and safety surveillance, but validated NF-specific thresholds are not yet available. Overall, Phase II management should be framed as infection surveillance plus evidence-bounded immune recovery, not routine immune stimulation.

### 6.3 Emerging adjunctive strategies and future directions

This section outlines non-standard adjunctive strategies that may provide incremental benefit in selected patients when used alongside foundational NF management: timely surgical source control, effective antimicrobial therapy, and comprehensive critical care support. The supporting evidence derives largely from observational studies, meta-analyses with substantial confounding risk, sepsis extrapolation, or preclinical models. Accordingly, these strategies should be interpreted cautiously, preferentially within experienced centers, clinical trials, or tightly governed investigational protocols, and must never delay definitive surgery, antimicrobial therapy, or organ support.

#### 6.3.1 Hyperbaric oxygen therapy

Hyperbaric oxygen therapy (HBOT) delivers 100% oxygen at elevated ambient pressure, increasing dissolved oxygen content and potentially raising tissue oxygen tension in hypoperfused regions. Mechanistically, HBOT has been proposed to inhibit or constrain obligate anaerobes, improve oxygen-dependent neutrophil killing, and partially counteract the perfusion-failure component of NF pathophysiology that limits antimicrobial delivery and immune-effector access.

Retrospective studies and meta-analyses associate HBOT with lower NSTI/NF mortality (Stevens et al., 2014; Hedetoft et al., 2021), but evidence certainty is low due to selection bias, center dependence, and confounding. Guidelines do not support routine HBOT, especially if it delays surgery or ICU resuscitation (Stevens et al., 2014; Huang et al., 2023). If used, it should follow urgent debridement, require transport stability and re-exploration readiness, and never delay source control.

#### 6.3.2 Extracorporeal cytokine removal and plasma exchange

Hemoperfusion or hemoadsorption approaches, most commonly cartridge-based cytokine adsorption, have been explored in sepsis and selected toxin-mediated syndromes as potential Phase I rescue adjuncts for extreme inflammatory mediator burden. In NF, the rationale would be strongest in fulminant hyperinflammation or toxin-driven shock after parallel initiation of source control, antitoxin antimicrobial therapy, and full organ support. However, controlled evidence remains limited and NF-specific trials are lacking.

A frequently cited randomized study of CytoSorb hemoadsorption in septic patients demonstrated increased IL-6 mass removal in the adsorption group but did not show a corresponding reduction in plasma IL-6 concentrations, and clinical outcome benefit was not established (Schädler et al., 2017; Padmanabhan et al., 2019). Therefore, routine cytokine adsorption cannot be recommended for NF outside clinical trials or tightly governed compassionate-use protocols. If attempted in exceptional circumstances, it should be framed as a time-limited, phenotype-triggered adjunct with predefined reassessment criteria.

Two safety principles are essential. First, adsorption devices may remove concomitant therapeutics, including antimicrobials, so antimicrobial exposure and dosing require careful attention. Second, nonspecific mediator removal may be inappropriate or harmful once an immunoparalysis-like state emerges, for example when sustained low mHLA-DR, lymphopenia, or impaired leukocyte responsiveness predominates. Plasma exchange has also been discussed as a strategy to remove circulating injurious factors while replacing protective plasma constituents. However, the broader severe-infection literature is heterogeneous and NF-specific clinical datasets are lacking. Plasma exchange should therefore not be positioned as a standard adjunct in NF and, if discussed, should be limited to research or highly selected investigational contexts with explicit inclusion criteria.

#### 6.3.3 Nutritional and metabolic support

Although not a single-target immunotherapy, nutritional and metabolic support is a cross-phase enabling intervention that supports immune competence, wound healing, and physiologic reserve. During prolonged ICU care, priority should be given to timely nutritional support, preferably enteral when feasible, adequate protein delivery, and correction of clinically relevant micronutrient deficiencies according to critical care nutrition principles. Glycemic management should avoid both severe hyperglycemia, which can impair neutrophil function and tissue repair, and overly aggressive strategies that increase hypoglycemia risk. In this sense, metabolic support should be viewed as an immune-supportive and repair-supportive foundation rather than an emerging immunomodulatory therapy.

#### 6.3.4 Integrating adjunctive strategies with immune phase and evidence boundaries

The potential value of any adjunctive strategy depends on timing, phenotype, and source-control status. Interventions that might be biologically plausible during one phase may be ineffective or harmful in another. For example, nonspecific anti-inflammatory or mediator-removal strategies could theoretically attenuate early inflammatory injury but may impair pathogen clearance or counteract immune recovery if applied during secondary immune dysfunction. Conversely, immune-restorative agents such as GM-CSF, IFN-γ, IL-7, or checkpoint-directed approaches may be rational only in biomarker-defined immunoparalysis-like states and could be inappropriate during uncontrolled hyperinflammation or persistent infection.

Therefore, adjunctive therapies in NF should be framed as evidence-bounded, phenotype-informed, and subordinate to the primary pillars of care: timely surgery, effective antimicrobial therapy, and comprehensive critical care support. Most current rationale is extrapolated from sepsis, invasive GAS, critical illness cohorts, observational NSTI studies, or preclinical models rather than adequately powered NF-specific randomized trials. A simplified phase-informed, phenotype-based overview of candidate adjuncts, evidence boundaries, and key cautions is provided in **Table 2**. Future progress will depend on prospective biomarker-stratified studies that integrate immune monitoring, tissue perfusion assessment, pathogen context, and source-control adequacy into trial design.

**Table 2:** Phase-informed, phenotype-based overview of adjunctive host-directed strategies in necrotizing fasciitis.

Immune state	Druggable goal	Adjunct option	Evidence boundary & caution
Phase I: toxin-amplified hyperinflammation (“Storm”)	Suppress toxin-driven amplification	β-lactam clindamycin (± linezolid)	•Best supported in GAS/STSS-oriented practice;
			•Do not delay debridement;
			•Monitor intolerance/resistance.
Phase I (STSS phenotype)	Neutralize circulating toxins	IVIG	•Evidence mixed/heterogeneous;
			•Consider only in severe toxin-driven/STSS features;
			•Infusion/thrombotic risks.
Phase I (hyperinflammation-enriched, MAS-like)	IL-1/NLRP3 axis modulation	Anakinra (IL-1 blockade)	•Mainly extrapolated (sepsis subgroup/preclinical);
			•Use only with biomarker-guided selection and stop rules.
Phase II: immune dysfunction/immunoparalysis (“Silence”)	Restore innate/adaptive function	GM-CSF/IFN-γ/IL-7	•Mostly sepsis immunoparalysis studies;
			•Risk of inflammatory rebound—require monitoring and predefined stop rules.
Phase II (T-cell exhaustion, exploratory)	Checkpoint reversal (PD-1/PD-L1)	Anti-PD-1/PD-L1	•Preclinical/early translational only; Immune-related adverse events;
			•Trial setting only.
Cross-phase (enabling adjunct; do not delay surgery)	Improve tissue oxygenation/perfusion	HBOT	•Evidence low certainty and center-dependent;
			•Use only if it does not delay source control/ICU care.
Cross-phase (rescue/protocol-based)	Mediator burden reduction (non-specific)	Hemoadsorption Plasma exchange	•Sepsis evidence mixed;
			•NF evidence minimal;
			•Protocolized rescue only;
			•Beware drug removal/bleeding.

## 7 Conclusions and future directions

NF is both a surgical emergency and a rapidly evolving host-response disorder in devitalized, hypoperfused tissue. Toxin- and pattern-recognition-amplified inflammation may overlap with counter-regulatory immune dysfunction across time and anatomical compartments, such that a persistently inflamed or pathogen-rich wound bed may coexist with peripheral immune deactivation, lymphopenia, or impaired antigen-presenting capacity. Outcomes depend on source control, antimicrobials including antitoxin agents when indicated, and critical care support. Once these foundations are secured, the translational challenge is to determine how serial host-response assessment can inform monitoring, source-control reassessment, infection surveillance, and evidence-bounded adjunctive evaluation.

### 7.1 Evidence gaps and limitations

NF immunopathogenesis and phase-informed adjuncts remain limited by rarity, retrospective single-center evidence, ICU-enriched cohorts, etiologic and host heterogeneity, and confounding from debridement timing, antimicrobials, organ support, and adjuncts. Immune-phase definitions are complicated by delayed presentation and uncertain disease onset. Peripheral immune markers may not capture fascial immunopathology, spatial gradients, or microvascular dysfunction. Future studies need prospective biomarker-stratified cohorts, standardized time-zero definitions, and, where feasible, spatial perfusion and immune assessments.

### 7.2 Immune profiling and phenotype-informed stratification

Future stratification should integrate immune phenotyping, functional biomarkers, pathogen features, and host determinants. Variables may include mHLA-DR trajectories, lymphocyte subsets, cytokines, leukocyte responsiveness, GAS toxin or immune-evasion programs, polymicrobial ecology, HLA background, immunoregulatory variation, immunometabolic constraints, barrier integrity, and microbiome context. Such schemes should move beyond broad labels and define testable host-response phenotypes for monitoring and adjunctive-care priorities.

### 7.3 Dynamic monitoring to define risk trajectories and therapeutic windows

Future strategies should move beyond static thresholds toward serial immune, inflammatory, and perfusion trajectories. Practical signals include lactate, CRP, procalcitonin, leukocyte and lymphocyte counts, and NLR, which are widely available and can support early trend-based reassessment. More direct immune monitoring, including mHLA-DR, lymphocyte subsets, cytokine panels, and ex vivo stimulation assays, may provide deeper functional information but will require assay standardization, clinically actionable turnaround times, locally validated interpretation, and NF-specific threshold development before routine bedside implementation. Spatial perfusion and viability assessment, including fluorescence-based mapping where available, may identify hypoperfused niches and support repeat-debridement decisions or trial eligibility.

### 7.4 Trial pathways for host-directed adjuncts

Host-directed adjuncts require prospective validation before routine use. Immune-restorative agents should be tested only in immune-dysfunction-enriched studies using endpoints such as sustained mHLA-DR depression, lymphopenia, or impaired leukocyte responsiveness. Antitoxin, anti-inflammatory, or complement-modulating approaches may suit toxin-dominant or complement-activated phenotypes. Given NF rarity, adaptive platform, biomarker-enriched, or basket-style protocols may be preferable to all-comer trials and must account for source control, pathogen context, perfusion, and secondary infection risk.

### 7.5 Toward earlier warning and spatially resolved assessment

Future progress in NF may depend on earlier risk stratification and more spatially resolved assessment of tissue perfusion and inflammatory activity. Multiplex biomarker panels, combined with transcriptomic or metabolomic signatures and accessible circulating markers of immunothrombosis, such as NET- and complement-associated fragments, may support early-warning approaches for identifying patients at risk of deterioration before overt shock or extensive necrosis develops. In parallel, emerging molecular imaging approaches, including probes for leukocyte activity, fibrin deposition, or thrombosis biology, and noninvasive tissue oxygenation mapping may support more localized assessment of tissue inflammation, perfusion failure, and viability. Together, these approaches could help integrate functional pathophysiology with conventional anatomic assessment and operative decision-making.

In summary, NF represents a catastrophic convergence of microbial virulence, tissue hypoperfusion, microvascular injury, and immune dysregulation. Reconceptualizing NF as a time-sensitive host-response disorder, while maintaining source control, antimicrobial therapy, and organ support as the foundations of care, may support more structured immune monitoring and phase-informed adjunctive evaluation. The next step is to determine prospectively whether biomarker-defined host-response trajectories can improve not only survival, but also limb salvage, wound-bed readiness, reconstructive success, secondary infection prevention, and long-term functional recovery.

## Glossary

APC: Antigen-presenting cell
CRP: C-reactive protein
CT: Computed tomography
DAMP: Damage-associated molecular pattern
GAS: Group A Streptococcus
G-CSF: Granulocyte colony-stimulating factor
GM-CSF: Granulocyte–macrophage colony-stimulating factor
HBOT: Hyperbaric oxygen therapy
HLA: Human leukocyte antigen
ICG: Indocyanine green
ICU: Intensive care unit
IFN: Interferon
IL: Interleukin
IVIG: Intravenous immunoglobulin
JAK: Janus kinase
LRINEC: Laboratory Risk Indicator for Necrotizing Fasciitis
MAPK: Mitogen-activated protein kinase
MAS: Macrophage activation syndrome
mHLA-DR: Monocytic human leukocyte antigen-DR
MHC: Major histocompatibility complex
MRI: Magnetic resonance imaging
MRSA: Methicillin-resistant Staphylococcus aureus
NADase: NAD-glycohydrolase
NET: Neutrophil extracellular trap
NF: Necrotizing fasciitis
NF-κB: Nuclear factor kappa B
NLR: Neutrophil-to-lymphocyte ratio
NLRP3: NOD-like receptor family pyrin domain containing 3
NSTI: Necrotizing soft-tissue infection
PAMP: Pathogen-associated molecular pattern
PCT: Procalcitonin
PD-1: Programmed cell death protein 1
PD-L1: Programmed death-ligand 1
PET/CT: Positron emission tomography/computed tomography
POCUS: Point-of-care ultrasound
PRR: Pattern-recognition receptor
ROS: Reactive oxygen species
SLO: Streptolysin O
SLS: Streptolysin S
STAT: Signal transducer and activator of transcription
STSS: Streptococcal toxic shock syndrome
TLR: Toll-like receptor
TNF: Tumor necrosis factor
